# Elucidation of the Active Agents in a West African Ground Herbal Medicine Formulation That Elicit Antimalarial Activities in In Vitro and In Vivo Models

**DOI:** 10.3390/molecules29235658

**Published:** 2024-11-29

**Authors:** Solomon Owumi, John O. Olanlokun, Bocheng Wu, Abiola Marian Duro-Ladipo, Sophia E. Oyelere, Shabana I. Khan, Adegboyega K. Oyelere

**Affiliations:** 1 Cancer Research and Molecular Biology Laboratories, University of Ibadan, Ibadan 200005, Nigeria; 2 Laboratories for Biomembrane and Biotechnology Research, Department of Biochemistry, University of Ibadan, Ibadan 200005, Nigeria; 3 School of Chemistry and Biochemistry, Georgia Institute of Technology, Atlanta, GA 30332, USA; bocheng.wu@gatech.edu; 4 Foetal Programming Unit, Department of Physiology, University of Ibadan, Ibadan 200005, Nigeria; abioladuroladipo@gmail.com; 5 Wheeler High School Magnet Program, Marietta, GA 30068, USA; sophiaeo4@gatech.edu; 6 NCNPR, School of Pharmacy, University of Mississippi, University, MS 38677, USA; skhan@olemiss.edu; 7 Parker H. Petit Institute for Bioengineering and Bioscience, Georgia Institute of Technology, Atlanta, GA 30332, USA

**Keywords:** *Alstonia boonei*, *Mangifera indica*, *Aristolochia repens*, *Enanthia chlorantha*, malaria, *Plasmodium berghei* (NK 65 and KI-37 strain), mitochondria

## Abstract

Agunmu (ground herbal medicine) is a form of West African traditional medicine consisting of a cocktail of herbs. The goal of this study is to evaluate a formulation of Agunmu made from *M. indica*, *A. repens*, *E. chlorantha*, *A. boonei*, and *B. ferruginea*, sold in the open market and commonly used for the treatment of malaria by the locals, for its antimalarial effects and to determine the active principles that may contribute to the antimalarial effect. The ethanolic extract obtained from this formulation (Ag-Iba) was analyzed, using TLC, LC-MS, and Tandem-MS techniques, to determine its phytochemical properties. The extract was tested in vitro against representative bacteria strains, cancer and normal human cell lines, and susceptible (D6) and resistant (W2) *Plasmodium falciparum*. In subsequent in vivo experiments, graded doses of the extract were used to treat mice infected with chloroquine-susceptible (NK-65) and chloroquine-resistant (ANKA) strains of *Plasmodium berghei*. Bacteria growth was monitored with a disc diffusion assay, cancer cell viability was determined with MTS assay, and percentage parasitemia and parasite clearance were determined by microscopy. Bound heme content, host mitochondria permeability transition (mPT) pore opening, F_0_F_1_-ATPase, and lipid peroxidation were determined via spectrophotometry. Indices of oxidative stress, anti-oxidant activities, toxicity, cell death, and inflammatory responses were obtained using biochemical and ELISA techniques. The histology of the liver and spleen was performed using the standard method. We elucidated the structures of the critical active principles in the extract to be flavonoids: kaempferol, quercetin, myricetin, and their glycosides with little or no detectable levels of the toxic Aristolochic acids that are found in *Aristolochia repens*, one of the components of the formulation. The extract also showed anti-plasmodial activity in in vitro and in vivo models. Furthermore, the extract dose-dependently decreased mitochondrial dysfunction, cell death, and inflammatory and oxidative damage but increased antioxidant potentials. Presumably, the active principles in the extract work as a combinatorial therapy to elicit potent antimalarial activity. Overall, our study unraveled the active components from a commercial herbal formulation that could be reformulated for antimalarial therapy.

## 1. Introduction

Malaria is a mosquito-borne disease caused by infection with four species of protozoan Plasmodium—*Plasmodium falciparum*, *P. malariae*, *P. ovale*, and *P. vivax*. Infections caused by the drug-resistant strains of these protozoans usually result in fatalities. The severity of malaria is more pronounced among pregnant women and children in sub-Saharan Africa, where access to conventional drugs for treatment and prevention is limited [1]. Studies have shown that liver and kidney failure are significant pathological events observed during uncomplicated and severe malaria [2,3]. These are manifested via dysregulations in the activities of some marker enzymes and abnormal concentrations of some blood proteins. The pre-erythrocytic stage of malarial infection in the liver causes damage to hepatocytes, with severe pathology at the organelle level. Mitochondrial pathology in malarial infection has also been reported [4,5]. During this infection period, there is an increase in hydroxyl radicals and a concomitant elevation of mitochondrial lipids’ peroxidation, causing oxidative stress [6].

Malarial pathogenesis involves damage carried out by the parasite on the critical organs of its hosts. Specifically, malarial pathogens affect the liver during the pre-erythrocytic stage. As a result, hepatocytes and organelles are adversely affected during this period. Furthermore, this disease can progress to the severe stage, causing liver failure, complications in the heart and kidney, severe anemia, and skeletal muscle dysfunction. At this stage of the disease, the patient’s hematological, cardiac, skeletal, and renal dysfunctions can be assessed using the serum levels of creatine kinase, troponins I and T, urea, and creatinine [7,8,9,10]. Unfortunately, some anti-malarial drugs cause discomfort related to malarial infection features as drug side effects. Previous studies have shown that some anti-malarial drugs cause lipid peroxidation [11,12], mitochondrial permeability transition (mPT) pore opening [12,13], and enhancement of F_1_F_0_ ATPase [12,14], as did the parasite infection [15,16]. The manifestation of some of these features has necessitated the withdrawal of some drugs. Furthermore, these side effects require searching for new anti-plasmodial medicines with minimal side effects and a broad spectrum of clearance of the malaria parasite burden.

Rural dwellers in Africa and Asia depend on phytomedicines to treat several diseases, including malaria. Agunmu (grounded herbal medicine) is one class of phytomedicines in West African traditional medicine practices. It is made from a cocktail of herbs pounded into a fine powder and commonly added to food [17]. Different cocktails of Agunmu exist for various indications, including inflammation, malaria fever (Agunmu-Iba (Ag-Iba), and bacterial and viral infections. The use of Ag-Iba to treat malaria is gaining wide recognition due to its low cost and demonstrable efficacy, especially against the drug-resistant strain of *P. falciparum* [5,17,18,19]. Although the Ag-Iba mechanism of action has not been fully elucidated, the individual medicinal plant component of Ag-Iba has been used to treat malaria. Usually, it exhibits antioxidant potentials [20,21], which help prevent oxidative stress, a common occurrence in malarial infection. The novelty of our study is underscored by the characterization of the critical active principles in the Ag-Iba cocktail comprising *Mangifera indica* L., *Aristolochia repens* (Mill), *Enantia chlorantha* (Oliv.), *Alstonia boonei* (De Wild), and *Bridelia ferruginea* (Benth.). The nomenclatures of these plants have been checked with “World Flora Online” www.worldfloraonline.org (accessed on 20 May 2024) (Table 1). The plants’ decoction (Ag-Iba) is used in West African traditional medicine to manage malaria. We also investigated the efficacy and toxicity profile of the Ag-Iba cocktail, following our proprietary extraction technique that reduces aristolochic acid. Each herb used in this Ag-Iba cocktail has been independently studied for anti-malarial effects. *Mangifera indica* [22], *Aristolochia repens* [23,24], *Enanthia chlorantha* [25,26], *Alstonia boonei* [5], and *Bridelia feruginea* [27] are primary herbs used separately as remedies for mild and complicated malaria. However, this folkloric cocktail’s anti-malarial properties, mitochondrial protective effect, antioxidant properties, capability to mitigate oxidative damage, and safety assessment have not been substantiated. Using LC-MS and MS/MS techniques, we found that the aqueous–alcoholic extract obtained from this Ag-Iba cocktail contained a mixture of kaempferol, quercetin, myricetin, and their glycosides with little or no detectable levels of the toxic Aristolochic acids that are found in *Aristolochia repens*, one of the components of the formulation. The extract elicits anti-plasmodial activities in in vitro and in vivo models and is less toxic to the liver and kidney.

## 2. Results

### 2.1. Analysis of the Ag-Iba Fractions

**Figure 1:**
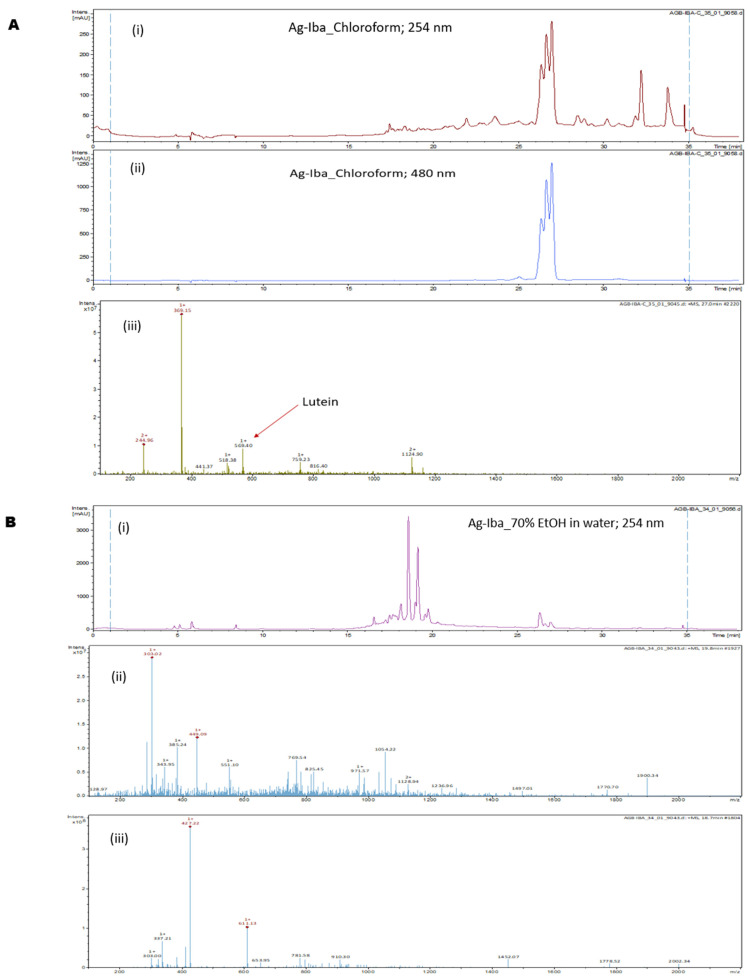
LC-MS analysis, monitoring at 254 nm and 480 nm, revealed that the xanthophyll carotenoids lutein and zeaxanthin are significant components of Ag-Iba CHCl_3_ extract (**A**(**i**–**iii**)). In contrast, Ag-Iba aqueous alcoholic extract has parent ion peaks at 303.02 [M + H]^+^, 449.09 [M + H]^+^, 595.15 [M + H]^+^, and 611.13 [M + H]^+^ (**B**(**i**–**iii**)). The positive ion mode is presented for clarity.

**Figure 2:**
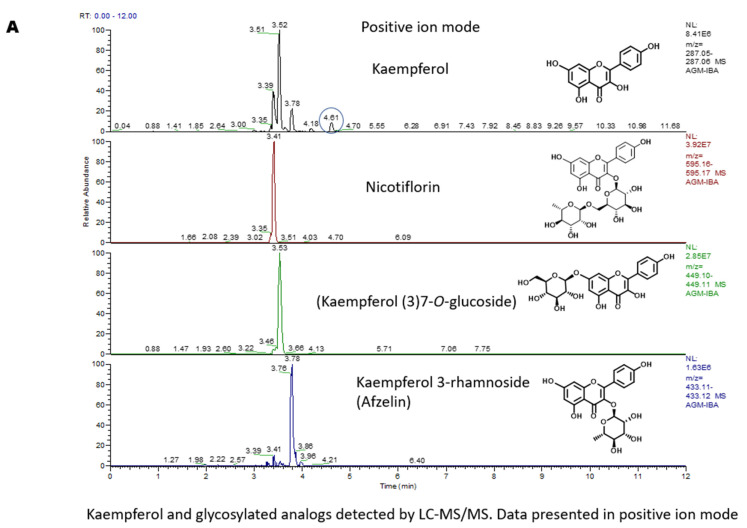

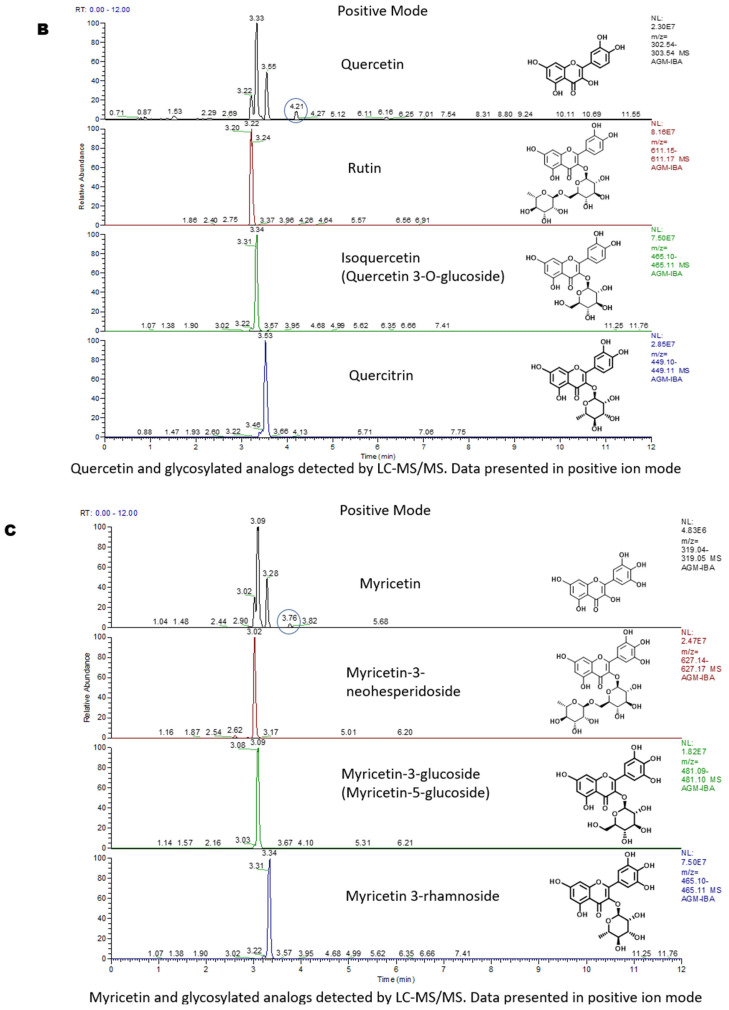
MS/MS analysis on the Ag-Iba aqueous alcoholic extract revealed the presence of (**A**) kaempferol and glycosylated analogs, (**B**) quercetin and glycosylated analogs, and (**C**) myricetin and glycosylated analogs. The data are presented in the positive ion mode.

We used the thin-layer chromatography (TLC), LC-MS, and MS/MS techniques to analyze the Ag-Iba CHCl_3_ and aqueous–ethanolic extract and identify the key components of the Ag-Iba fractions. TLC on a normal phase plate using CHCl_3_/MeOH 18:1 showed that the Ag-Iba CHCl_3_ extract components clustered into three spots with retention factors (Rf) of 0.34, 0.64, and 0.85, in addition to minor components close to the solvent front and those tailing on the TLC plate (Figure S1A). The TLC of the Ag-Iba aqueous alcoholic extract in CHCl_3_/MeOH 18:1 showed that it contained (a) major constituent(s) that remained at the baseline and other spots with an Rf of 0.17 and 0.34 (Figure S1B). Subsequently, we independently analyzed the two extracts by LC-MS monitoring at 254 nm and 480 nm. For the Ag-Iba CHCl_3_ extract, we observed that the absorption maxima (λ_max_) drastically increased at 480 nm relative to 254 nm, and a peak with molecular weight 569.4 [M + H]^+^ (retention time = 26.6 to 27.0 min) was detected (Figure 1A(i–iii)). The preceding finding suggested that this extract is enriched in a mixture of xanthophyll carotenoids lutein and zeaxanthin. Although the Ag-Iba aqueous alcoholic extract has a minor component showing a similar absorption spectrum characteristic to the Ag-Iba CHCl_3_ extract, its major components are absorbed at 254 nm but not 480 nm. The key parent peaks detected were 303.02 [M + H]^+^, 449.09 [M + H]^+^, 595.15 [M + H]^+^, and 611.13 [M + H]^+^ (Figure 1B(i–iii)). Subsequent rigorous MS/MS analysis revealed that the Ag-Iba aqueous alcoholic extract contained a mixture of flavonoids putatively identified as predominantly kaempferol, quercetin, myricetin and their glycosides including afzelin, nicotiflorin, kaempferol-3-O- or Kaempferol-7-O-glucoside, rutin, isoquercetin, quercitrin, myricetin-3-neohesperidoside, myricetin-3-O- or myricetin-5-O-glucoside, and myricetin 3-rhamnoside. The identities of the representative compounds kaempferol, quercetin, myricetin, and rutin were confirmed using standards (Figure 2A–C and Figure S2A–D). TLC analysis of the alcoholic extract, relative to the Kaempferol and myricetin standards, in CH_2_Cl_2_/MeOH 12:1, revealed that the extract contained a component with the same Rf as Kaempferol (0.45), while there is a tailing close to the myricetin Rf (0.20) in the extract as well (Figure S1C).

### 2.2. Ag-Iba Aqueous Alcoholic Extract Elicits an Anti-Plasmodial Effect but Is Devoid of Anti-Bacterial Activity and Not Cytotoxic to the Tested Eukaryotic Cell Lines

To obtain information about the diversity of the biological activities of the alcoholic extract of Ag-Iba, we screened for its effect on the proliferation of two representative bacterial strains, four representative transformed and non-transformed eukaryotic cells, and two strains of plasmodium species. We observed that the extract, at concentrations as high as 33 mg/mL (5 μL and 10 μL), has no effect on the growth of *B. subtilis* (Gram-positive) or *E. carotovora* (Gram-negative), as evidenced by a lack of zone of inhibition in the disc diffusion assay (Figure S3A). Similarly, Ag-Iba aqueous alcoholic extract has no significant deleterious effect on the viability of A549, MCF-7, Hep-G2, and Vero cells up to 1 mg/mL (Figure S3B). Interestingly, the Ag-Iba aqueous alcoholic extract elicited an anti-malarial effect by suppressing the proliferation of the W2 strain of plasmodium with IC_50_ of 43.8 μg/mL. At the same time, its IC_50_ against the D6 strain was undetermined at the maximum tested concentration of 47.6 mg/mL (Table 2).

### 2.3. Ag-Iba Aqueous Alcoholic Extract Dose-Dependently Decreased Erythrocyte Parasite Load, Increased Parasite Clearance, and Heme Content

The blood and liver cells, especially the erythrocytes and hepatocytes, respectively, are the most affected in malarial parasite infection. We monitored this parasite’s effect on blood and liver tissue, especially liver mitochondria, an energy-generating organelle.

**Figure 3:**
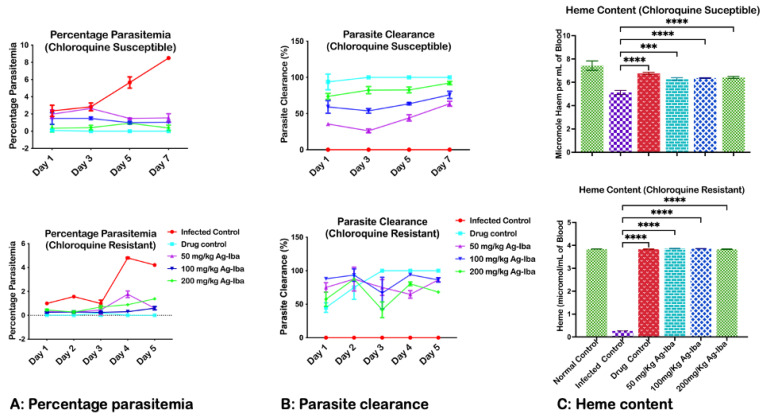
Effects of the herbal mixture on percentage parasitemia (**A**), parasite clearance (**B**), and heme content (**C**) in chloroquine-susceptible and chloroquine-resistant strains of *Plasmodium berghei*. The NK 65 and ANKA strains of *Plasmodium berghei* were separately used to induce malaria in mice. The susceptible and resistant experiments were monitored for seven and five days, respectively. Microscopy was performed to estimate the percentage of parasitemia (**A**) and parasite clearance (**B**). The bound heme content for the two experiments (**C**) was performed to show the extent of hemolytic anemia. The experiments were performed thrice, and the data are expressed as mean values ± SD. *** = *p* < 0.001, **** = *p* < 0.0001 test groups vs. infected control.

Malarial parasite load increased in erythrocytes when not treated but decreased with a sufficient therapeutic drug dose. In this case, this herbal combination reduced the parasite load dose-dependently, indicating that the solvent system extracts potent phytochemicals from the herbal mixture. Specifically, on day 7 of the phase I experiment, there was no significant difference between the percentage parasitemia value of the standard drug and the highest dose (200 mg/kg) of the herbal mixture (Figure 3A). Consequently, the parasite clearance (chemo-suppression) increased dose-dependently (Figure 3B), and there was no significant difference between the chemo-suppressive effect of the standard drug and the herbal cocktail. Similar results were observed in the phase II experiment, where the curative potentials of Ag-Iba were determined against mice infected with chloroquine-resistant *Plasmodium berghei.* It was also observed that Ag-Iba decreased the parasite load over five days against the increase in the parasite load in the infected control (Figure 3C). Correspondingly, the parasite clearance increased over five days (Figure 3B). The determination of heme content showed that the herbal mixture improved the treated group’s heme content. Although the heme content of the infected groups treated with the herbal mix decreased (*p* < 0.0001) when compared with a standard control, there was no significant difference between the heme content of the infected mice treated with 100 and 200 mg/kg of the herbal mixture and the standard drug. However, there was a 50 mg/kg decrease (*p* < 0.05) in mice infected with chloroquine-sensitive and chloroquine-resistant strains of *Plasmodium berghei* (Figure 3C).

### 2.4. Ag-Iba Aqueous Alcoholic Extract Reverses Mitochondrial Permeability Transition Pore Opening and the Enhancement of Mitochondrial F_0_F_1_ ATPase Activity

**Figure 4:**
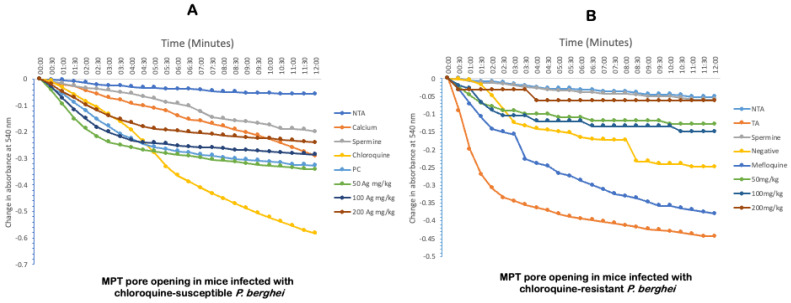
The kinetic effects of the herbal mixture on mitochondrial permeability transition pores (MPT). These figures depict the extent of liver mitochondria isolated from the infected control response to permeabilization due to *Plasmodium berghei* infection. They also show that the reversal effect of the administered drug leads to parasite-induced permeabilization in mice infected with chloroquine-susceptible (**A**) and chloroquine-resistant (**B**) strains of *Plasmodium berghei.* Normal mitochondria without Triggering Agent (NTA); normal mitochondria with Triggering Agent (TA)—calcium; parasitized control (PC) without any treatment.

To further understand the effect of malarial infection on hepatocytes in the pre-erythrocytic stage, we determined the effect of malarial infection on mitochondria via its pore opening effects and the mitigating effect of the herbal mixture (Figure 4). Here, significant amplitude swelling was noticed as an increase in the decrease in the absorbance. Interestingly, calcium, an established pore inducer, opened the mitochondrial pore with a large amplitude (five folds). Furthermore, chloroquine opened the pore further with a significant amplitude swelling to nine folds. In Figure 4A, it is interesting to note that the anti-malarial herbal mixture reversed the pore opening dose-dependently. The 50, 100, and 200 mg/kg reversed the mitochondrial pore opening up to six folds (50 mg/kg), five folds (100 mg/kg), and four folds (200 mg/kg). Similar results were obtained in the resistant experiment. Here, 200 mg/kg of Ag-Iba reversed the parasite-induced opening of the pore as much as the standard inhibitor. Chloroquine and mefloquine, the standard drugs for treating malaria in chloroquine-sensitive and chloroquine-resistant experiments, respectively, caused a significant amplitude swelling of mitochondria and opening of the mitochondrial permeability transition in pores (Figure 4B).

### 2.5. Ag-Iba Aqueous Alcoholic Extract Mediated the Enhancement of ATPase Activity in Both Studies of Plasmodium Parasite Infection

**Figure 5:**
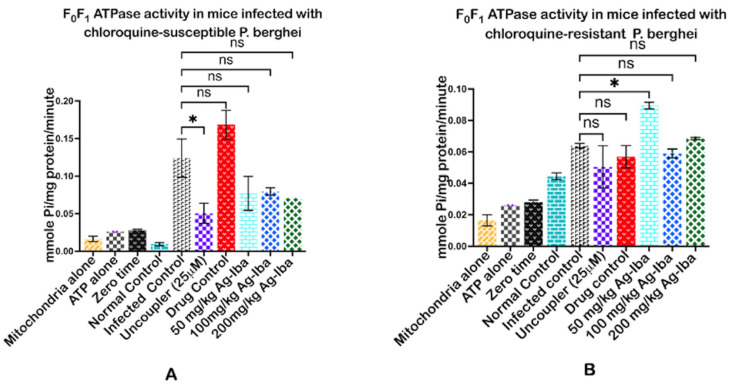
The influence of the herbal mixture on the enzymatic hydrolysis of ATP via the activity of mitochondrial F_1_F_0_ ATPase in mice infected by the chloroquine-susceptible (**A**) and chloroquine-resistant (**B**) *Plasmodium berghei.* Minimal activity of the enzyme in mitochondria is only evidence that the organelles were intact and remained uncoupled. Similarly, the minimal level of the inorganic phosphate released in ATP only showed that the substrate (ATP) was well kept and did not undergo hydrolysis by heat before assay. A low level of released inorganic phosphate at zero time showed that ATP hydrolysis by the enzyme is time-dependent. The experiments were performed thrice, and the data were expressed as mean values ± SD. * = *p* < 0.05 test groups vs. infected control, while ns means insignificant.

To further corroborate this herbal mixture’s pore opening reversal effect, we determined its effect on F_0_F_1_ ATPase. We observed that both infected and positive controls significantly (*p* < 0.01, 0.001, respectively) enhanced mitochondrial F_0_F_1_ ATPase activity. At the same time, the herbal mixture dose-dependently decreased the activity of this enzyme in the chloroquine-sensitive model. In the infected and drug control group, the activity of the ATPase enzyme was significantly enhanced, more than what was observed in the uncoupler group (Figure 5A). Furthermore, in mice infected with the resistant *Plasmodium* parasite, the herbal mixture could not considerably reverse the enhancement of mitochondrial F_0_F_1_ ATPase activity relative to the infected and drug controls. The 50 mg/kg dose of the composite herbal preparation significantly (*p* < 0.05) enhanced the activity of the ATPase enzyme more than the infected control (Figure 5B).

### 2.6. Ag-Iba Aqueous Alcoholic Extract Decreases Aspartate, Alanine Aminotransferases, and Alkaline Phosphatase

**Figure 6:**
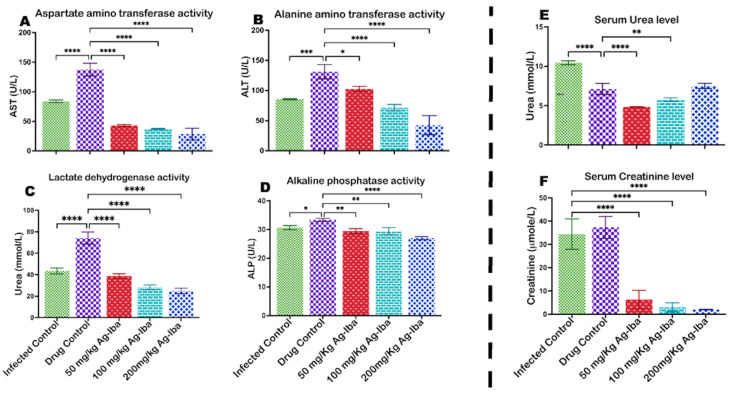
Effects of the herbal mixture on Aspartate aminotransferases (**A**), alanine aminotransferases (**B**), lactate dehydrogenase (**C**), and alkaline phosphate (**D**) as marker enzymes for the hepatotoxicity of the herbal preparation, while serum urea (**E**) and creatinine (**F**) levels were used to assess the renal toxicity of the herbal preparation. The experiments were performed thrice, and the data are expressed as mean values ± SD. * = *p <* 0.05; ** = *p* < 0.01; *** = *p <* 0.001; **** = *p* < 0.0001 controls vs. test groups.

Since the pre-erythrocytic stage of *Plasmodium* infection affects the liver and this same organ is the site for biotransformation of drugs and drug candidates, we further evaluated the effects of this infection and drug on the liver cells via the activities of aspartate, alanine aminotransferases (AST, ALT), lactate dehydrogenase (LDH), and alkaline phosphatase (ALP). It was observed that chloroquine increased the levels of these enzymes when used to treat malaria (Figure 6A–D). Malaria infection also increases the activities of these enzymes. However, the herbal preparation used in this study decreased (*p* < 0.05) the activities of AST (Figure 6A), ALT (Figure 6B), LDH (Figure 6C), and alkaline phosphatase (Figure 6D).

### 2.7. Ag-Iba Aqueous Alcoholic Extract Protects the Kidney by Regulating Serum Urea and Creatinine Levels

Urea and creatinine are by-products of the protein and tissue metabolism used to assess kidney excretory prowess. In this study, urea and creatinine levels in mice infected with *Plasmodium berghei* but not treated significantly increased, as shown in Figure 6E,F. The herbal mixture used to treat malaria significantly decreased serum urea and creatinine levels relative to the positive control. Specifically, creatinine level decreased dose-dependently (Figure 6F), while the level of urea slightly increased (though insignificantly) when compared with the positive control (Figure 6E).

### 2.8. The Effect of Ag-Iba Aqueous Alcoholic Extract on the Superoxide Dismutase, Catalase, and Glutathione Peroxidase Activity Reduced Oxidative Stress Indices

**Figure 7:**
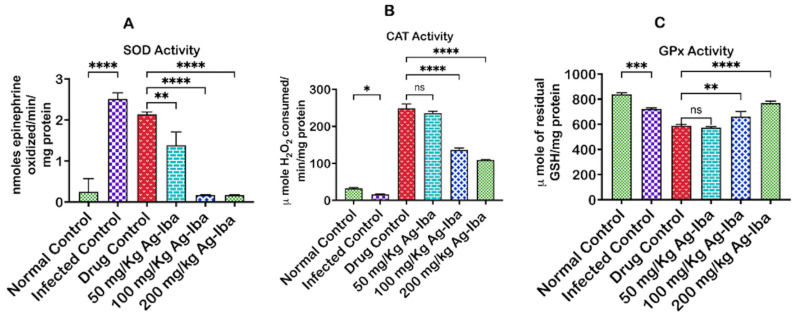
Effects of the herbal mixture on superoxide dismutase (SOD) (**A**), catalase (**B**), and glutathione peroxidase (GPx) (**C**) activities in *Plasmodium berghei*-infected mice. The experiments were performed thrice, and the data are expressed as mean values ± SD. Not significant (ns); * = *p* < 0.05; ** = *p* < 0.01; *** = *p <* 0.001; **** = *p* < 0.0001 comparisons between the mean values of the test groups and controls.

Figure 7 depicts the effect of the Ag-Iba herbal mixture on some antioxidant parameters. We observed an increase (*p* < 0.0001) in the activities of superoxide dismutase (SOD) in the infected control relative to the standard control, indicating that *Plasmodium berghei* infection caused an increase in superoxide radicals’ generation in the experimental mice (Figure 7A). It was also observed that an increase in the activity of catalase (CAT) in the drug control indicated that this drug’s mechanism of action may be related to the generation of free radicals, as is the case with many anti-malarial drugs (Figure 7B). Interestingly, Ag-Iba dose-dependently decreased the activity of these enzymes (SOD and CAT) in the infected mice treated with the drug preparation. Glutathione peroxidase (GPx) is an intracellular antioxidant enzyme that reduces hydrogen peroxide to water to limit its peroxidative and harmful effects. *Plasmodium berghei* infection causes a decrease (*p* < 0.001) in GPx activity compared with the standard control (Figure 7C). Although there was no significant difference in the activity of this enzyme in the infected mice treated with the standard drug and the lowest dose of Ag-Iba (50 mg/kg), there was a corresponding increase in GPx activity due to increasing the doses of Ag-Iba.

### 2.9. Ag-Iba Aqueous Alcoholic Extract Abated Oxidative-Stress-Mediated Damages and Improved Glutathione-Dependent Enzyme Utilization

**Figure 8:**
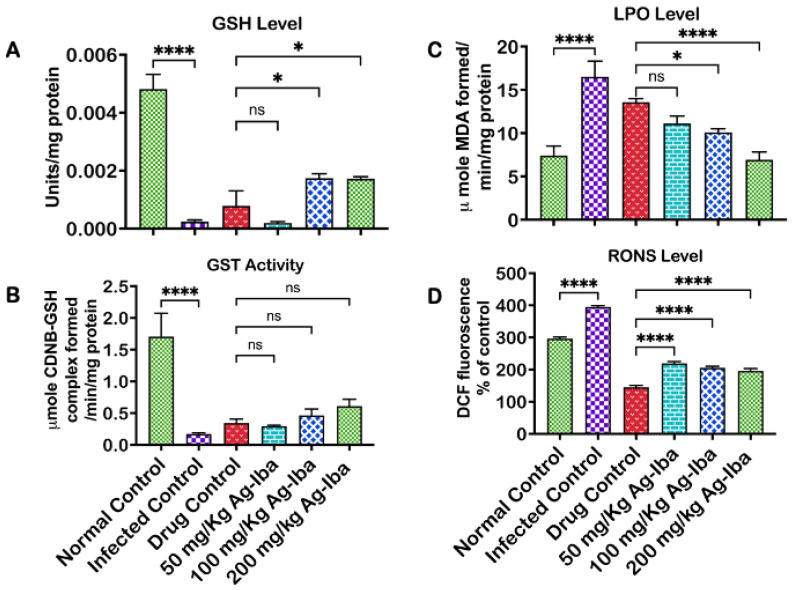
Effects of the herbal mixture on glutathione (**A**), glutathione-dependent enzyme (**B**), lipid peroxidation (**C**), and reactive oxygen and nitrogen species (**D**). The experiments were performed thrice, and the data are expressed as mean values ± SD. * = *p* < 0.05, **** = *p* < 0.0001 comparison between the mean values of the test groups and controls; ns means not significant.

The effect of the Ag-Iba herbal mixture on GSH/GST profile, lipid peroxide formation, and RONS production is depicted in Figure 8. *Plasmodium* infection can cause reduced glutathione depletion, which can restore the antioxidant capacity of a central antioxidant defensive mechanism, glutathione-*S*-transferase (GST). Infection by *Plasmodium berghei* depleted (*p* < 0.0001) the serum concentration of the reduced glutathione when compared with the standard control. However, the treatment of mouse malaria with Ag-Iba increased (*p* < 0.05) the serum glutathione level compared with the standard drug or infected controls (Figure 8A). Serum glutathione-*S*-transferase activity was the highest in the standard control un-infected by *Plasmodium berghei*, and the least activity was observed in the infected control. In contrast, appreciable GST activity was observed in the serum of infected mice treated with the standard drug and graded doses of Ag-Iba, albeit insignificantly (Figure 8B). There was an increase (*p* < 0.0001) in the serum level of thiobarbituric reactive substances (TBARSs) in the infected but not treated mice compared with the normal control. Treatment with the standard drug decreased the serum levels of TBARS. Furthermore, a dose-dependent decrease in TBARS was observed when infected mice were treated with Ag-Iba (Figure 8C).

In response to *Plasmodium* infection, phagocytes such as macrophages and neutrophils generate reactive oxygen and nitrogen species (RONS) as a natural defensive mechanism, as observed in the infected control in this study compared with the standard control (*p* < 0.0001) (Figure 8D). However, when the parasite burden decreases, the levels of these reactive species decrease because of the effectiveness and potency of the administered drug. The administered standard drug reduced (*p* < 0.0001) the serum level of these reactive species compared to all the doses of Ag-Iba used in this study.

### 2.10. Ag-Iba Aqueous Alcoholic Extract Abated Pro-Inflammatory and Apoptotic Biomarker Responses in Experimental Mice

**Figure 9:**
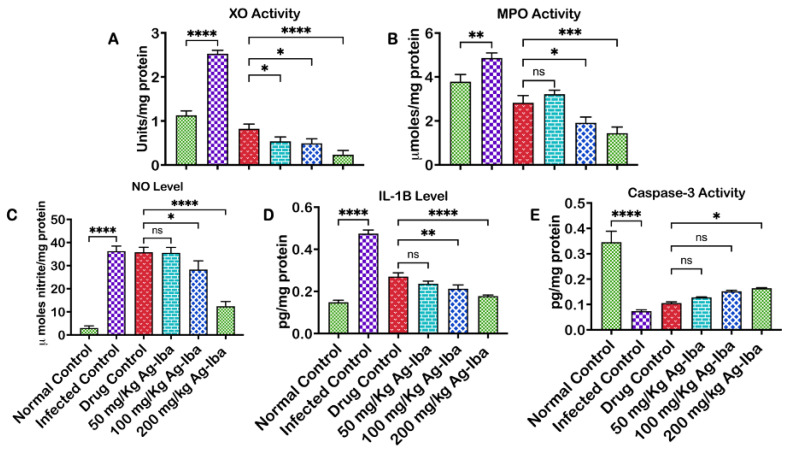
Effects of the Ag-Iba herbal mixture on biomarkers of oxidative stress using xanthine oxidase (XO) (**A**), myeloperoxidase (MPO) (**B**) activities; inflammation nitric oxide (NO) (**C**), and interleukin-1β) (**D**) levels, and apoptosis via the activity of the executioner caspase (caspase-3) (**E**). The experiments were performed thrice, and the data are expressed as mean values ± SD. * = *p <* 0.05, ** = *p <* 0.01; *** = *p <* 0.001; **** = *p* < 0.0001 among the test groups and controls; ns means not significant.

Oxidative damage, inflammation, and cell death are common secondary effects of *Plasmodium* infection. This experiment was carried out to investigate, in addition to a decrease in or total obliteration of the parasite load, the efficacy of Ag-Iba on the secondary effects of *Plasmodium* infection. The impact of the Ag-Iba on biomarkers relevant to inflammation and apoptosis is depicted in Figure 9. Xanthine oxidase activity increased (*p* < 0.0001) in the infected control by the *Plasmodium berghei* infection, and the standard drug decreased the observed increase in XO activity. However, Ag-Iba further reduced the activity of this enzyme dose-dependently and maximally (*p* < 0.0001) at 200 mg/kg (Figure 9A). Myeloperoxidase activity, a biomarker of neutrophil activation, usually occurs as one of the defense mechanisms against malaria infection. A similar effect was noticed on the activity of myeloperoxidase activity (*p* < 0.001 relative to the drug control) at the highest dose of Ag-Iba (Figure 9B). As expected, parasite load activates the increase in the level of this enzyme. However, the administration of drugs decreased the serum activity of this enzyme, and the maximal effect was noticed when Ag-Iba was administered at 200 mg/kg to infected mice compared with when the standard drug was used (*p* < 0.001). In Figure 9C, *Plasmodium* infection caused an increase in the serum level of nitric oxide, and this increase was gradually reduced when the graded dose of Ag-Iba was administered to the infected mice.

Moreover, a maximum decrease was observed when Ag-Iba (200 mg/kg) was administered to infected mice. Furthermore, there was an increase in the serum level of IL-1β, a pro-inflammatory marker in the infected control. The serum level of IL-1β was comparatively decreased in infected mice treated with the highest dose of Ag-Iba (Figure 9D). It was observed that there was a decrease in the activity of caspase 3, an executioner caspase, in the infected control and that administration of the drugs caused an increase in this caspase activity, albeit insignificantly (Figure 9E).

### 2.11. Histology of the Liver and Spleen Did Not Show Severe Lesions When Infected Mice Were Treated with Ag-Iba Aqueous Alcoholic Extract

**Figure 10:**
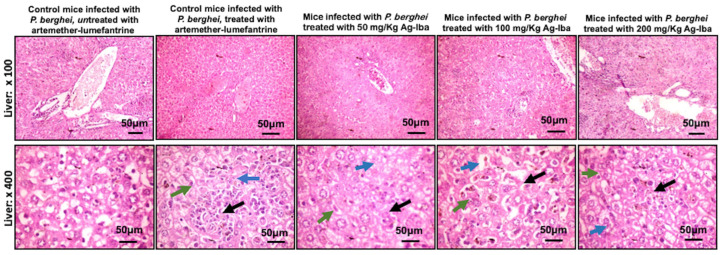
Plates showing the histology of the liver of the infected control, infected control treated with a standard antimalaria drug (artemether-lumefantrine), and infected mice treated with 50, 100 and 200 mg/kg doses of Agunmu-Iba (Ag-Iba).

The liver of the normal un-infected mice showed moderate disseminated congestion (blue arrows), mild microvesicular steatosis (green arrows), and the liver of the infected control showed mild to moderate disseminated periportal infiltration by inflammatory cells (black arrows) and moderate disseminated infiltration of zone 2 by inflammatory cells (black arrows) Figure 10. The liver of mice treated with the control drug showed moderate disseminated congestion/thrombosis (blue arrows) and mild microvesicular steatosis (green arrows). In contrast, those treated with 50, 100, and 200 mg/kg of Ag-Iba showed mild macrovesicular steatosis (green arrows), mild to moderate disseminated periportal infiltration by inflammatory cells, mild macrovesicular steatosis (green arrows), mild to moderate disseminated periportal infiltration by inflammatory cells and moderate disseminated infiltration of zone 2 by inflammatory cells (black arrows) and moderate disseminated congestion (blue arrows), mild macrovesicular steatosis (green arrows), and mild to moderate disseminated periportal infiltration by inflammatory cells and moderate disseminated infiltration of zone 2 by inflammatory cells (slender arrows), respectively.

**Figure 11:**
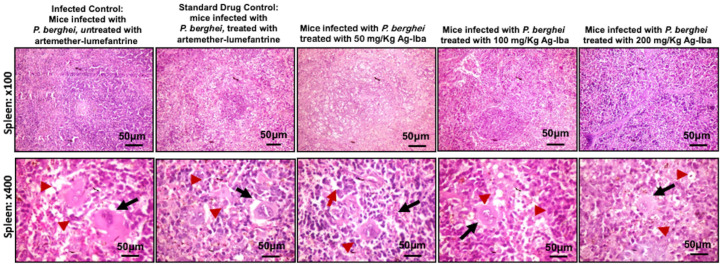
Plates showing the histology of the spleen of the infected control, infected control, treated with a standard antimalaria drug (artemether-lumefantrine), and infected mice treated with 50, 100 and 200 mg/kg doses of Agunmu-Iba (Ag-Iba). Lesion in the section of the spleen are indicated by red arrows.

The normal control mice’s spleen showed well-delineated white and red pulp, and numerous megakaryocytes (black arrows) are in the marginal zone of the red pulp (red arrowhead; Figure 11). Mice treated with the control drug showed well-delineated white pulp and red pulp. There are numerous megakaryocytes (black arrows) in the marginal zone of the red pulp (red arrowhead). Whereas mice treated with 50, 100, and 200 mg/kg of Ag-Iba showed well-delineated white pulp and red pulp, with numerous megakaryocytes in the marginal zone of the red pulp (red arrowhead) (Ag-Iba 50 mg/kg. Well-delineated white and red pulp, where also observed in the 100 mg/kg Ag-Iba treated mice characterised with several megakaryocytes in the marginal zone of the red pulp (black arrows). Finally, in the 200 mg/kg Ag-Iba treated mice similar trend was observed, characterised by well-defined zones of white and red pulp, with abundant megakaryocytes (black arrows) in the marginal zone of the red pulp (red arrowhead).

## 3. Discussion

Infection by strains of *Plasmodium* that are resistant to orthodox drugs is a severe health challenge, often accompanied by increased fatality. There are few orthodox therapeutic options for patients infected with the drug-resistant plasmodium strains, necessitating the search for alternative curative, low-cost drug candidates such as medicinal plants. Phytomedicines used for therapy by the populace in malaria-endemic areas of Africa and Asia often contained combinations of medicinal plants. These combinations are informed by experience gathered over several millennia. The medicinal plant cocktails offer a promising source of new single agents or combination therapy regimens for the treatment of malaria. One phytomedicine cocktail in West Africa formulated for various diseases, including malaria, is Agunmu (ground herbal medicine). Due to its low cost and the evidence of its efficacy, especially against the drug-resistant strain of *P. falciparum*, the use of Agunmu-Iba (Ag-Iba) to treat malaria is gaining traction. However, the mechanisms of action of Ag-Iba cocktails have not been fully elucidated. In this study, we characterized the predominant active principles and investigated the efficacy and toxicity profile of an aqueous alcoholic extract of Ag-Iba cocktail comprising *Mangifera iundica*, *Aristolochia repens*, *Enanthia chlorantha*, *Alstonia boonei*, and *Bridelia feruginea*.

The LC-MS trace of the CHCl_3_ extract of Ag-Iba revealed that it is enriched in a mixture of xanthophyll carotenoids lutein and zeaxanthin. Although the aqueous–alcoholic extract has a minor component showing a similar spectra characteristic as the Ag-Iba CHCl_3_ extract, it predominantly contained a mixture of the flavonoids kaempferol, quercetin, myricetin, and their glycosides. Several of these molecules have been reported to have anti-malarial activities [28,29,30,31,32,33,34]. Therefore, this study was focused on the Ag-Iba aqueous alcoholic extract.

The Ag-Iba aqueous alcoholic extract is not broadly cytotoxic as it did not elicit a toxic effect against the prokaryotic and eukaryotic cells tested. However, it is selectively cytotoxic to the W2 strain of plasmodium with IC_50_ of 43.8 μg/mL. While we could not determine the IC_50_ of the extract against the D6 strain at the maximum concentration tested (47.6 mg/mL), it may likely elicit cytotoxicity against the D6 stain at higher doses. This Ag-Iba aqueous alcoholic extract possibly derives *Plasmodium* cytotoxicity from its flavonoid constituents, some known to possess independent anti-malarial properties. However, the biological basis of this selectivity remains unclear. A plausible major contributing factor is the ability of some of these flavonoids to generate ROS, which induces oxidative damage, and the *Plasmodium* parasites are poorly adapted for it [35,36]. Given that the Ag-Iba aqueous alcoholic extract is non-toxic against the eukaryotic cells tested, another exciting possibility is that this extract will elicit minimal systemic toxicity in vivo. To investigate this prospect, we tested the extract in vivo using murine models of susceptible and resistant strains of *P. berghei*.

We evaluated the infection and parasite clearance of various doses of the Ag-Iba aqueous alcoholic extract using microscopy [37]. We observed that the extract has promising anti-malarial potential against both susceptible and resistant strains of *Plasmodium* infection. A more significant percentage of the erythrocytes in the thin blood smears from the infected control animals were invaded by *P. berghei*. All the randomly viewed fields in the experimental animal erythrocyte slides reflect this. However, we observed a dose-dependent decline in the parasitemia in the erythrocytes of treated animals. Furthermore, as treatment progressed, there was a decrease in the parasite load, corresponding to the dose of the Ag-Iba. Interestingly, this herbal mixture has chemo-suppressive effects on the resistant strain of *P. berghei*.

The antiplasmodial effects elicited by the Ag-Iba aqueous alcoholic extract in this rodent model make the decoction of *M. indica* [38], *A. species* [39], *E. chlorantha* [40], *A. boonei* [4,41], and *B. ferruginea* [27] a promising source to identify candidates for novel combinational therapy for malaria. The percentage of parasite suppression in infected mice treated with *M. indica* (62.20% at 200 mg/kg), *A. indica* (52.30% at 300 mg/kg), *E. chlorantha* (66.60% at 400 mg/kg), *A. boonei* (78.00% at 200 mg/kg), and *B. feruginea* (69.97% at 200 mg/kg) was by far less when compared with a composite formulation of these medicinal plants both in the susceptible (90.50%) and resistant (80.00%) strains used in these studies. It is possible that the combination of these herbs was better than that of single medicines because of their synergistic effects in clearing the parasite load, preventing complications such as oxidative stress and anemia due to their antioxidant potentials [6,42]. Bound heme in the erythrocytes is responsible for transporting oxygen and carbon dioxide through the bloodstream. Thus, heme is critical for oxidative metabolism, including oxidative phosphorylation. Bound heme decreases in the erythrocytes of infected mice via hemolysis. An increase in the concentration of the bound heme in the infected but treated mice indicated that the Ag-Iba prevents the hemolysis of the erythrocyte membrane. The harmful effect of *Plasmodium* infection is felt in the red blood cell where the parasites take charge of the host cell machinery, breaking down heme for its metabolic purpose. A decrease in the heme content of erythrocytes is linked to anemia and is one of the complications of *Plasmodium* infection [43,44].

Interestingly, the Ag-Iba aqueous ethanolic extract significantly increased the cellular heme content, similar to the standard antimalarial drug (positive control), further corroborating Ag-Iba’s antiplasmodial potentials. Indirectly, a decrease in heme catabolism may be accountable for reducing hemozoin content.

In the pre-erythrocytic stage of *Plasmodium* infection, the liver cells and organelles are adversely affected by direct insults on the cells by the parasites or because of oxidative stress. Mitochondria is a major organelle directly affected by *Plasmodium* infection, affecting mitochondrial metabolism, including ATP generation. Previous studies have shown that malaria can cause mitochondrial-mediated cell death through the opening of the mitochondrial pore [18] or the translocation of Bax from the cytosol to the outer mitochondrial membrane to oligomerize with Bak [45]. Furthermore, a study has shown that orthodox anti-malarial drugs caused mitochondrial membrane permeabilization [12]. However, the Ag-Iba aqueous ethanolic extract reversed mPT pore opening caused by the *Plasmodium* infection in both models (susceptible and resistant), as observed in a previous study [46]. Ag-Iba phytochemicals may block the site for calcium and phosphate interactions (both inducers of the pore) and render them unavailable for binding to initiate mPT pore opening. In addition, oxidative stress could likely exacerbate the permeabilization of the outer mitochondrial membrane, which can also lead to cell death [47]. However, Ag-Iba’s inherent antioxidants could help scavenge free radicals responsible for this effect. The idea that antioxidants reverse mitochondrial pore opening has been reported [48].

The mitochondrial ATP synthase is a bi-directional enzyme that synthesizes and hydrolyze ATP (as ATPase) to compensate for a decrease in mitochondrial membrane potential. The modulation of ATP synthase is central to the integrity of the mitochondrion and critical to the lifespan of some organisms [49]. We observed that ATPase activity decreased both in the susceptible and resistant models, showing that Ag-Iba can protect the mitochondria, having reversed mitochondrial pore opening and reducing the cytosolic concentration of Pi, known as an inducer of mPT. It also increases the enzyme’s activity as an ATP synthase but decreases its hydrolysis and, as a result, maintains a balance in ATP metabolism.

The liver is central in the pre-erythrocytic stage of *Plasmodium*’s life cycle and is seriously affected during this stage in untreated malaria. Loss of function and outright hepatic failure have been reported in acute and complicated malaria [50,51]. Alanine and aspartate aminotransferases (ALT and AST) are marker enzymes for liver integrity. An increase in the serum activity of these enzymes, observed in the infected control, may indicate hepatocyte damage and enzyme leakage into the serum. ALT and AST serum activities were reduced in infected mice treated with Ag-Iba in both models, suggesting that the Ag-Iba preserves the liver ultra-structure. There are different ways by which the Ag-Iba could have elicited this protective effect. Hepatic disorders could have occurred because of infection [52]. Ag-Iba flavonoid-based active principles could have been hepato-protective via the inhibition of inflammation [53,54], immunomodulation [55], the inhibition of lipid peroxidation, and decreased calcium influx implicated in cell death [56].

Furthermore, the generation of ROS in malaria and the imbalance in the oxidant/antioxidant ratio cause oxidative stress implicated in hepatocyte damage [6]. The decrease in the activities of superoxide dismutase and catalase enzymes may be traceable to the low ROS levels in the infected mice treated with the Ag-Iba, underscoring its antioxidant effects. However, it is known that the activities of these enzymes are high in the infected and drug (artemether–lumefantrine) controls. Since *Plasmodium* infection liberates iron (ferrous) from the digested hemoglobin, the mechanism of action of some anti-malarial drugs is via free radical generation [6,57,58,59,60]. Increased glutathione peroxidase activity, sustained by an increase in the serum levels of reduced glutathione, will enhance the reduction of lipid peroxides, which are by-products of peroxidation of membrane phospholipids, as observed in this study. The reduction in the serum RONS of infected mice treated with Ag-Iba shows that Ag-Iba can stem the tide of the pathophysiological effects of *Plasmodium* infection.

Xanthine oxidase activity is increased in malarial disease, further exacerbating the inflammatory response. Only an effective anti-malaria treatment might mitigate this effect [61]. The observed increase in the level of serum interleukin-1β in the infected mice corroborates this premise, since interleukin-1β is an essential mediator of inflammation [62]. Nitric oxide (NO) levels have been implicated in malaria pathology [63], with high levels of serum NO reported in infected control [64]. High serum NO is correlated to a high plasma level of arginase, possibly with concurrent hypoargininemia, indicative of the innate protective mechanisms against malaria because previous studies have shown that NO protects against malaria [65,66]. Increased myeloperoxidase activity is a marker of increased neutrophil activation in malaria disease; therefore, an increase in granulocyte number in peripheral blood is observed when coupled with leukocyte hemozoin accumulation [67]. Apoptotic cell death in the liver or eryptosis in the blood cells is more common in infected cells, and it is a mechanism by which the parasite load is reduced [68]. Comparatively, Ag-Iba dose-dependently increased apoptosis, which may correlate with a reduced parasite load. Some of the constituents of this herbal mixture had earlier been reported to have antioxidant potential. Therefore, the herbal mixture could have protected the liver by reducing the intracellular quantity of reactive oxygen species by enhancing the hepatocytes’ enzymic activities and non-enzymic antioxidant levels [69].

Alkaline phosphatase (ALP) is an enzyme in many body parts but can primarily be found in the liver, intestines, and kidneys. The serum level of ALP is a leading biomarker of hepatobiliary injury, such as cholestatic liver injury. Quinine was the initial drug used for the prophylactic treatment of malaria. However, despite its effectiveness as a chemo-preventive agent for malaria, quinine and chloroquine, its aminoquinolone analog, cause hepatic injury. Furthermore, amodiaquine, a structural analog of chloroquine, is an effective anti-malarial, but it causes severe hepatitis and agranulocytosis [70]. It is interesting, however, that a drug from medicinal plants could have such efficacy against *Plasmodium* infection and yet manifest little or no toxicity through reduced serum alkaline phosphate levels.

Previous studies have detected *Plasmodium* antigens in the glomeruli, suggesting that *Plasmodium* infection can directly affect the kidney and thus trigger the inflammatory process, leading to glomerulonephritis and affecting kidney function. Although currently out of use because of their ineffectiveness against the resistant *Plasmodium* species, the long-term use of chloroquine [71], and quinine [72,73] has been linked with renal injury. Other clinical manifestations of the involvement of the kidney in malaria disease include proteinuria and albuminuria. These effects may further show an increase in serum urea and creatinine. We further observed that serum urea and creatinine levels in the infected control mice group and those infected but treated with the drug control increased (Figure 4A,B), contrary to what was observed in the serum levels of urea and creatinine of mice infected with *Plasmodium berghei* but treated with Ag-Iba. The preceding observations imply that Ag-Iba also has a protective effect on the kidney and its anti-plasmodial effect.

Some herbal decoctions, when not used moderately, are hepatotoxic or nephrotoxic. For instance, *Aristolochia* species contain Aristolochic acids that cause kidney failure and other health complications [74]. The danger of *Aristolochia* species has been recognized, and their use as components of herbal medicine has been prohibited in the US, some Western countries, and Japan [75,76]. Nevertheless, *Aristolochia* species remain actively used in herbal medicines in Africa and some parts of Asia. Gratifyingly, an analysis of our MS data revealed that the abundance of peaks that may be attributed to Aristolochic acid I (negative mode, calc: 340.0463, observed: 340.0430) and Aristolochic acid II (negative mode, calc: 310.0357, observed: 310.0606—less reliable due to >80 ppm deviation from the expected peak) within the noise level. The lack of toxicity of the aqueous–alcoholic extract of the grounded herbal medicine we used in this study to VERO cells (Figure S3B) eases the fear of nephrotoxicity, one of the immediate toxic effects of Aristolochic acids. Others have noted that extraction methods could lower the Aristolochic acid contents of extracts from *Aristolochia* species [77]. So, it is highly likely that our extraction enriched the antimalarial flavonoid components of the grounded herbal medicine while minimizing the Aristolochic acid level. Moreover, the histology of the liver and spleen showed that the herbal decoction was less toxic as there were no severe lesions in the spleen or the liver of the treated infected mice. Furthermore, data obtained from the biochemical tests for toxicity (Figure 4) coupled with the histology of the liver and spleen (Figure 8 and Figure 9) showed that the doses used in this study are safe. Steatosis in the standard control and some intervention groups showed that the diet is fatty and could be responsible for fat accumulation. There are reports of steatosis, which may not be a severe concern, in previous studies [78]. The megakaryocytes noticed in the spleen of normal control mice are platelet-synthesizing cells [79], and this could occur in infection because platelets have been previously reported to kill *Plasmodium* parasites [80].

## 4. Materials and Methods

### 4.1. Collection of Plant Materials

The medicinal plants, fresh stem barks of *Alstonia boonei*, *Aristolochia repens*, *Bridelia feruginea*, *Enanthia chlorantha*, *and Mangifera indica*, were purchased from the Medicinal Herbs Section of the Bodija Market, Ibadan, Nigeria. Geographically, the Bodija market lies between longitude 3°54′36″ E and 3°55′12″ E and latitude 7°25′52″ N and 7°26′22″ N [81]. Plant specimens were identified and authenticated by Mr. F. O. Omotayo of Plant Herbarium, Plant Science Department, Ekiti State University, Nigeria, and voucher numbers UHAE 2024021, 2024022, 2024023, 2024024, and 2024025 (respectively) were assigned to the plant samples.

### 4.2. Extraction and Characterization of Phytochemicals in Ag-Iba

Ag-Iba (20 g) was suspended in CHCl_3_ (150 mL) and stirred for 24 h. The suspension was filtered, and the filtrate was evaporated to give the CHCl_3_ extract a greenish foam (0.78 g). The Ag-Iba after CHCl_3_ extraction was air-dried, suspended in 70% EtOH in water (150 mL), and stirred at room temperature for 24 h. The suspension was filtered, and the filtrate was evaporated to give the Ag-Iba aqueous alcoholic extract as a brownish solid (1.42 g).

### 4.3. The Liquid Chromatography–Mass Spectrophotometry (LC-MS) Analysis of Ag-Iba Extract

The Ag-Iba aqueous alcoholic and CHCl_3_ extracts were analyzed by LC-MS monitoring at 254 nm and 480 nm on a Bruker amaZon SL ion trap mass spectrometer coupled to an Agilent 1260 HPLC (Agilent Technologies, Santa Clara, CA, USA). Chromatography was performed on a Phenomenex C18 reversed-phase HPLC column (250 × 4.6 mm; S/NO: H17-238,591) (Torrance, CA, USA), as we have described previously. Mass spectrometry data were collected in the positive and negative ionization modes in the mass range *m*/*z* 100–1000 Da.

### 4.4. Tandem Mass Spectrometry (MS/MS) Analysis

High-resolution mass spectra and MS/MS were recorded at the Georgia Institute of Technology Mass Spectrometry Facility.

### 4.5. In Vitro Anti-Bacterial, Anti-Cancer and Anti-Plasmodium Assays

The potential anti-bacterial activity of the Ag-Iba aqueous alcoholic extract was evaluated against *B. subtilis* (Gram-positive) and *E. carotovora* (Gram-negative) using the agar disc diffusion assay [82]. As we described before, the MTS assay was used to evaluate the effect of the extract on the proliferation of representative lung (A549), breast (MCF-7), and liver (Hep-G2) cancer cell lines and a non-transformed cell (Vero). The in vitro antiplasmodial activity of the Ag-Iba aqueous alcoholic extract was evaluated against two strains of *P. falciparum*, namely D6 (chloroquine sensitive) and W2 (chloroquine-resistant). The assay is based on measuring plasmodial lactate dehydrogenase (LDH) activity according to Makler and Hinrichs’s procedure [83]. In brief, a 200 µL suspension of red blood cells in RPMI 1640 medium supplemented with 10% human serum and 60 µg/mL amikacin, infected with *P. falciparum* (2% parasitemia and 2% hematocrit), is added to the wells of a 96-well plate containing serially diluted samples (10 µL each). The plate was incubated in a modular incubation chamber under the following gaseous tension: N_2_ (95%), O_2_ (5%), and CO_2_ (5%) at 37 °C for 72 h. To measure the parasitic LDH activity, 20 µL of the incubation mixture was transferred to another plate mixed with Malstat reagent (100 µL) and incubated at 37 °C for 30 min. Subsequently, 20 µL of nitrotetrazolium blue chloride and phenazine ethosulfate (NBT/PES; 1:1 solution) were added. The plate was incubated further for one hour while protected from the direct light source. The reaction was stopped by adding 5% acetic acid solution (100 µL). At 650 nm, the reaction absorbance was measured. The test samples’ percentage reduction in growth inhibition was computed and contrasted with the vehicle controls. In addition, primaquine, artemisinin, and chloroquine were included as the drug controls. Finally, the growth inhibition dose–response curves were used to calculate the IC_50_ values.

### 4.6. Experimental Animals, Infection, and Treatment Protocols

#### Curative Effect of the Aqueous–Alcoholic Extract Ag-Iba on Chloroquine-Susceptible (NK 65) Strain of Plasmodium Berghei-Infected Mice

A modified method of Ryley and Peters [84] to establish the infection protocol was used for this study in two phases: in the first phase, a chloroquine-sensitive (NK 65) strain of *Plasmodium berghei* was used to infect the mice, while for the second phase, a chloroquine-resistant (ANKA) strain was used to infect the mice. In the first phase, twenty-five male Swiss mice (15 ± 2 g) were obtained from the animal house unit of the Malaria Research Section Institute of Advanced Medical, Research and Training, College of Medicine, University of Ibadan, Nigeria. The experimental mice were acclimatized for two weeks in the Department of Biochemistry, University of Ibadan, and were fed with standard rat chow and access to water ad libitum. Malaria was later induced via a single intraperitoneal injection of infected erythrocytes (10^7^ inoculum) obtained from a donor mouse. Parasitemia was confirmed via microscopy after 72 h, and they were grouped (n = 5) as follows:

***Group 1:*** Infected mice but not treated (10 mL/kg of the vehicle);

***Group 2:*** Infected mice but treated with the control drug (10 mg/kg chloroquine);

***Group 3:*** Infected mice treated with 50 mg/kg of the Ag-Iba herbal combination;

***Group 4:*** Infected mice treated with 100 mg/kg of the Ag-Iba herbal combination;

***Group 5:*** Infected mice treated with 200 mg/kg of the Ag-Iba herbal combination.

Another set of mice (five) was used as the standard un-infected control but fed with normal rat chow. The animals were treated for seven days, and slides were collected at two-day intervals. The second phase was similar to the first but with some differences: the same number and groups of animals were used. However, the Swiss mice were infected with the chloroquine-resistant (ANKA) strain of *Plasmodium berghei.* After the confirmation of parasitemia, mice were treated for five consecutive days, and blood slides were prepared and stained daily for microscopy.

### 4.7. Treatment, Slide Preparation, and Microscopy

Grouped infected mice were treated orally and once daily with graded doses of the extract. A 10 mL/kg (10% dimethyl sulfoxide (DMSO)) sample and 10 mg/kg of the vehicle and standard drugs (chloroquine for the sensitive strain and mefloquine for the resistant strain) were each orally administered once daily. The test groups were treated with graded doses (50–200 mg/kg) of Agunmu-Iba. After a series of pilot studies, the authors arrived at these doses with minimum and maximum doses without toxic effects (unpublished data). Since no activity occurred after inoculation until parasitemia was confirmed after 72 h and treatment commenced, we refer to the day that treatment commenced as day 1 for the chloroquine-sensitive and resistant studies. Percentage parasitemia and parasite clearance were determined by microscopy. Slides were collected via the tail snip, and the blood smears were prepared on the slides as thin films. Collected thin-film slides were air-dried and fixed with absolute methanol. The fixed slides were arranged on slide racks and stained with a Giemsa (10% stock) solution for twenty-five minutes. After that, the slides were rinsed with buffered water and air-dried. Stained slides were viewed under a binocular microscope; first, they were focused using the ×10 objective, and later, three independent fields per slide were viewed and counted using the ×100 objective (oil immersion). The percentage parasitemia and parasite clearance were calculated as follows:

$$\mathrm{Percentage}\;\mathrm{Parasitemia}=\frac{\mathrm{Number}\;\mathrm{of}\;\mathrm{infected}\;\mathrm{erythrocytes}\;\mathrm{counted}\;\times \;100}{\mathrm{Total}\;\mathrm{erythrocytes}\;\left(\mathrm{infected}\;\mathrm{and}\;\mathrm{non}-\mathrm{infected}\right)}$$

$$\mathrm{Percentage}\;\mathrm{clearance}=\frac{\mathrm{Counted}\;\mathrm{infected}\;\mathrm{erythrocytes}\;\left(\mathrm{control}\right)-\mathrm{Counted}\;\mathrm{infected}\;\mathrm{erythrocytes}\;\left(\mathrm{Test}\right)\times 100}{\mathrm{Counted}\;\mathrm{infected}\;\mathrm{erythrocytes}\;\left(\mathrm{control}\right)}$$

### 4.8. Heme Content Determination

The total heme content was estimated when the animals were terminated to provide information on the oxygen-carrying capacity of the red blood cells. This was carried out using blood withdrawn from experimental animals using the established method of Asakura et al. [85]. Briefly, 10 µL of whole blood was mixed with 250 µL 10% SDS (*w*/*v*) and 250 µL of 1 M NaOH, followed by sonication for 10 min. The tubes were incubated for two hours at room temperature, and the total heme content was determined in a spectrophotometer at 404 nm. Assuming the molar absorption coefficient of heme to be 9.08 × 10^4^/M/cm, the heme concentration was expressed as mmol of heme/mL of blood.

### 4.9. Isolation of Mitochondria from the Experimental Mice

Mitochondria were isolated using the method of Johnson and Lardy [86]. After the days of treatment, the animals were terminated via cervical dislocation and quickly opened, and the liver was removed, rinsed with isolation buffer (210 mM Mannitol, 70 mM Sucrose, 5 mM Hepes-KOH (pH 7.4) and 1 mM EGTA) to remove blood stains, and weighed. It was later chopped. A 10% suspension was homogenized at 4 °C, loaded into a centrifuge (Sigma 300k, Hamburg, Germany) at the same temperature, and spun at 2500 rpm twice at 5 min to sediment cell debris and unbroken cells. The supernatant was spun for 10 min at 12,000 rpm to pellet mitochondria. Mitochondria were washed twice at 10,000 rpm each time for 10 min using the washing buffer (210 mM Mannitol, 70 mM Sucrose, 5 mM Hepes-KOH (pH 7.4) and 0.5% BSA) and later suspended in suspension buffer (210 mM Mannitol, 70 mM Sucrose, 5 mM Hepes-KOH (pH 7.4). Washed mitochondria were later dispensed in aliquots into Eppendorf tubes and kept on ice. Mitochondria used for F_0_F_1_ ATPase activity were isolated using a similar procedure, but all buffers were substituted with 0.25 M sucrose.

### 4.10. Mitochondrial Protein Determination

Mitochondrial protein was determined by using the conventional Lowry et al. method [87]. In triplicates, 10 µL mitochondria were added to distilled water, and the volume was brought up to 1 mL with distilled water. Later, 3 mL of a 100:1:1 ratio mixture of 2 g Na_2_CO_3_ and 1% CuSO_4_·5H_2_O in 0.1 M NaOH was added to each test tube, and the mixture was incubated at room temperature for 10 min. A five-fold dilution of Folin reagent stock solution (0.3 mL) was added, and the mixture was vortexed and incubated at room temperature for 30 min. The absorbance was read at 750 nm. Mitochondrial protein content was determined from a standard curve prepared using BSA.

### 4.11. Assessment of Mitochondrial Permeability Transition

The evaluation of isolated mitochondrial integrity for mitochondrial permeability transition (mPT) pore opening was determined using Lapidus and Sokolove [88], with modifications. Isolated mitochondrial protein (0.4 mg/mL) from the standard control was incubated in a suspension buffer containing 8 µM rotenone for 3.5 min, then 5 mM succinate was added, and the absorbance was read for 12 min at 30 s intervals to ascertain the uncoupled status of the mitochondria. The susceptibility to calcium was observed by incubating the same mitochondrial protein in a suspension buffer containing rotenone for 3 min, and then 3 µM CaCl_2_ was added. Thirty seconds later, 5 mM sodium succinate was added, and absorbance was read. The inhibitor, spermine, was used to reverse calcium-induced mPT opening by incubating the same mitochondrial protein in suspension buffer, rotenone, and 4 mM spermine for 3 min before adding calcium. Succinate was added 30 s later, and absorbance was read. When mitochondria have a minor change in absorbance in the absence of calcium, but mPT is induced in the presence of calcium, which is highly reversed by spermine, such a mitochondrial isolate is adjudged to be uncoupled and suitable for mPT study. Equivalent mitochondrial protein from the treated groups was subjected to the same permeability transition under the same condition.

### 4.12. Estimation of Mitochondrial F_0_F_1_ ATPase Activity

Mitochondrial F_0_F**_1_** ATPase activity was assessed as described by Lardy and Wellman [89]. Reagents (25 mM sucrose, 65 mM Tris-HCl (pH 7.4), and 0.5 mM KCl) were added into test tubes, and the volume was brought up to 1 mL. Adenosine triphosphate (1 mM) was added to tubes designated “ATP only”, “uncoupler”, “zero-time”, “test groups”, and “controls” tubes, while “mitochondria only” tubes did not contain ATP. These were transferred to the shaking water bath and incubated at 27 °C. Mitochondria (0.5 mg/mL protein) isolated from the livers of mice from the different groups were added to their designated test tubes. A set of test tubes labeled “mitochondria-only” contained the above reagents and mitochondria isolated from the normal control. This ensured that the F_0_F**_1_** enzyme was not previously activated, leading to a false negative result. Another set of test tubes labeled “uncoupler” contained 25 µM of 2,4-dinitrophenol used as the standard control. As mentioned, the last set of test tubes labeled “zero-time” contained mitochondria isolated from the normal control group and the reagents. Sodium dodecyl sulfate (SDS) 1 mL was immediately added to stop the reaction, which ensured that the release of inorganic phosphate from ATP was time-dependent. The last set of test tubes labeled “ATP only” contained the above reagents and the substrate, ATP. This ensures that the substrate had not been hydrolyzed before adding mitochondria. Other sets of test tubes contained the reagents, mitochondria protein from the treated groups and ATP, as substrate. These test tubes were incubated at 25 °C in a shaker Waterbath for 30 min. After incubation, the reaction was stopped using SDS in all the tubes (except the zero-time test tubes that had been stopped earlier) with 1 mL SDS. The test tubes were taken out, and 1 mL of the reaction mixture was sampled into a separate test tube and diluted with distilled water (4 mL). Ammonium molybdate (1.25%; prepared in 6.5% H_2_SO_4_) and freshly prepared ascorbic acid (9%), 1 mL each, were added, and the absorbance at 660 nm was taken spectrophotometrically. A standard phosphate curve was used to calculate the amount of inorganic phosphate released.

### 4.13. Analysis of the Antioxidant Potentials of Ag-Iba

Biochemically, serum activities of superoxide dismutase (SOD: Misra and Fridovich method) [90], catalase (CAT, following Clairborne’s method) [91], glutathione peroxidase (GPx, according to the method of Rotruck et al.) [92], reduced glutathione (GSH, according to Beutler) [93], and glutathione-*S*-transferase activity (GST, Habig’s method), as well as lipid peroxidation (LPO, according to the method of Okhawa et al.) [94], and reactive oxygen and nitrogen species (RONS, according to the method of Perez-Severiano et al.) [95], were determined. The serum activities of the enzymes and levels of the peroxidative and reactive species products were determined by following standard biochemical methods as previously reported.

### 4.14. Determining the Effects of Ag-Iba on Oxidative Damage, Inflammation, and Cell Death Assays

Markers of oxidative damage such as xanthine oxidase (XO) and myeloperoxidase (MPO) activities were determined following standard laboratory protocols, as previously described and reported [96,97,98]. Furthermore, biomarkers of inflammation—interleukin 1β (IL-1β) level and apoptosis executioner caspase (caspase-3)—activity were determined by Enzyme-Linked Immunosorbent Assay (ELISA) kits purchased from Elabscience (Wuhan, China). IL-1β levels and caspase-3 activity were determined by closely following the detailed manufacturers’ protocols.

### 4.15. Assessment of Biomarkers of Liver and Kidney Functions from Experimental Mouse Serum

This experiment was carried out for the resistant model only. Analyses of aspartate aminotransferase (AST), alanine aminotransferase (ALT), alkaline phosphatase (ALP), and lactate dehydrogenase (LDH), as well as creatine and urea levels, were performed using available commercial kits from Randox ^TM^ Laboratories Limited (Crumlin, UK) and a Molecular Devices SpectraMax 384^TM^ multi-modal microplate reader (San Jose, CA, USA).

### 4.16. Histology of Liver and Spleen

Liver and spleen histology was performed using the standard procedure to show lesions in these tissues emanating from *Plasmodium berghei* infection or because of treatment.

The experimental mouse tissues of interest were sectioned into small pieces (<4 mm thick) and placed in properly labeled cassettes. The tissues were fixed by immersion in formal saline (10%) for 24 h. The fixed tissue was processed automatically using a Leica TP 1020 tissue processor by passage through stations 1 and 2 containing various reagents, including 10% formal saline. Station 3 through 7: dehydration by alcohol (70%, 80%, 90%, 95%, absolute I and absolute II). This was followed by two changes of xylene cleaning via Stations 8 and 9. Finally, the tissues were transferred thrice into wax baths for infiltration/impregnation. The automatic tissue processor was programmed for a 12 h run, during which the tissues being processed stayed in each station for an hour. Each processed tissue was supported in a medium of paraffin wax, achieved by a semi-automatic tissue embedding center. The tissue was buried for coating in molten paraffin wax and pre-dispensed into a metal mold, which was allowed to solidify and was labeled and separated from the mold. Subsequently, the tissue blocks were trimmed to approximately 6 μm and allowed to cool on ice before sectioning to 4 μm (ribbon section). The sections were floated in a water bath at 55 °C and placed on clean slides. The slides were labeled and allowed to dry on a hotplate set at 60 °C for an hour. The slides were stained with hematoxylin and eosin staining techniques.

### 4.17. Statistical Analysis

Representative profiles of the absorbance of mitochondria kinetically measured at 540 nm were used for mitochondrial permeability transition. The endpoint determination of other experiments was performed in triplicates. These data are represented as mean ± standard deviation. The statistical and significance analyses of data were performed by comparing the control data with the test groups using the one-way analysis of variance with GraphPad Prism (v.9.0) for MacOS and Tukey’s post hoc multiple comparison method.

## 5. Conclusions

In conclusion, we showed herein that Ag-Iba contains phytochemicals that elicit potent antimalarial effects against chloroquine-sensitive and -resistant strains of *Plasmodium berghei* in murine models. Furthermore, Ag-Iba reverses mitochondrial permeability transition pores that could cause mitochondrial dysfunction and, by extension, toxicity to the cells. Our data suggest that Aristolochic acids, and consequently the *Aristolochia repens* component of the herbal formulation used in this study, contributed negligibly to the antimalarial activity of the formulation. So it is likely that a formulation lacking *Aristolochia repens*, which will avoid the harmful health consequences of the consumption of this herb, will work as well. Alternatively, the active principles in the extract that work as a combinatorial therapy to elicit potent antimalarial activity could be reformulated for antimalarial treatment. We are investigating the anti-malarial activities of the reconstituted components that closely mimicked the ratios we observed in the herbal extract. Findings from these future studies will be reported in a follow-up report.

## Figures and Tables

**Table 1:** Constituents of Agumu–Iba for managing malaria infection.

Common Names	Local (Yoruba) Names	Botanical Names
1. Mango bark	Mangoro	*Mangifera indica* L.
2. Dutchman’s pipe	Ako igun	*Aristolochia repens* (Mill)
3. African yellow wood bark	Awopa or Yaani	*Enantia chlorantha* (Oliv.)
4. Stool wood leaves and bark	Ahun	*Alstonia boonei* (De Wild)
5. Unknown	Ira bark	*Bridelia ferruginea* (Benth.)

The nomenclatures of the plants were verified (20 May 2024) with “World Flora Online” www.worldfloraonline.org.

**Table 2:** In vitro antimalarial activity of Ag-Iba aqueous alcoholic extract based on measuring plasmodial lactate dehydrogenase (LDH) activity.

	D6 Strain	W2 Strain
Samples	IC50 μg/mL	IC50 μg/mL
Ag-Iba	NA *	43.8
Primaquine	0.3	0.4
Chloroquine	0.014	0.2
Artemisinin	0.0053	0.0088

* No activities up to the highest concentration of 47.6 μg.

## Data Availability

The data presented in this study are available on request from the corresponding author due to potential issues with the patent.

## Supplementary Materials

The following supporting information can be downloaded at: https://www.mdpi.com/article/10.3390/molecules29235658/s1, Figure S1: TLC analysis of the chloroform (A) and aqueous alcoholic extract (B and C) of Ag-Iba. TLC in CH_2_Cl_2_/MeOH 12:1 revealed that the aqueous–alcoholic extract contained a component with the same Rf as kaempferol (0.45) (C). Figure S2: (A) Confirmation of kaempferol: LC (top) and MS/MS (bottom) revealed that the sample and standard have the same retention time and fragmentation pattern. (B) Confirmation of quercetin: LC (top) and MS/MS (bottom) revealed that the sample and standard have the same retention time and fragmentation pattern. (C) Confirmation of myricetin: LC (top) and MS/MS (bottom) revealed that the sample and standard have the same retention time and fragmentation pattern. (D) Confirmation of rutin: LC (top) and MS/MS (bottom) revealed that the sample and standard have the same retention time and fragmentation pattern. Figure S3: (A) Disc diffusion assay. Lack of zone of inhibition in the disc diffusion assay revealed that Ag-Iba aqueous alcoholic extract is devoid of anti-bacterial activity. (B) MTS assay revealed that Ag-Iba aqueous alcoholic extract has no significant deleterious effect on the viability of A549, MCF-7, Hep-G2, and Vero cells up to 1 mg/mL.

## References

[1] Imboumy-Limoukou R.K., Maghendji-Nzondo S., Sir-Ondo-Enguier P.N., Niemczura De Carvalho J., Tsafack-Tegomo N.P., Buekens J., Okouga A.P., Mouinga-Ondeme A., Kwedy Nolna S., Lekana-Douki J.B. (2020). Malaria in children and women of childbearing age: Infection prevalence, knowledge and use of malaria prevention tools in the province of Nyanga, Gabon. Malar. J..

[2] Reuling I.J., de Jong G.M., Yap X.Z., Asghar M., Walk J., van de Schans L.A., Koelewijn R., Farnert A., de Mast Q., van der Ven A.J. (2018). Liver Injury in Uncomplicated Malaria is an Overlooked Phenomenon: An Observational Study. EBioMedicine.

[3] Junior G.B.S., Pinto J.R., Barros E.J.G., Farias G.M.N., Daher E.D.F. (2017). Kidney involvement in malaria: An update. Rev. Inst. Med. Trop. Sao Paulo.

[4] Olanlokun J.O., Okoro P.O., Lawal O.S., Bodede O., Olotu F., Idowu T.O., Prinsloo G., Soliman M.E., Olorunsogo O.O. (2021). Betulinic acid purified from *Alstonia boonei* inhibits folate biosynthesis in malarial *Plasmodium*, enhances mitochondrial pore opening and F1F0 ATPase in mice. J. Mol. Struct..

[5] Olanlokun J.O., Bolaji O.M., Agbedahunsi J.M., Olorunsogo O.O. (2012). Therapeutic effects of various solvent fractions of *Alstonia boonei* (apocynaceae) stem bark on *Plasmodium berghei*-induced malaria. Afr. J. Med. Sci..

[6] Percario S., Moreira D.R., Gomes B.A., Ferreira M.E., Goncalves A.C., Laurindo P.S., Vilhena T.C., Dolabela M.F., Green M.D. (2012). Oxidative stress in malaria. Int. J. Mol. Sci..

[7] Salazar J.H. (2014). Overview of Urea and Creatinine. Lab. Med..

[8] Plewes K., Turner G.D.H., Dondorp A.M. (2018). Pathophysiology, clinical presentation, and treatment of coma and acute kidney injury complicating falciparum malaria. Curr. Opin. Infect. Dis..

[9] Akinosoglou K.S., Solomou E.E., Gogos C.A. (2012). Malaria: A haematological disease. Hematology.

[10] Davis T.M., Pongponratan E., Supanaranond W., Pukrittayakamee S., Helliwell T., Holloway P., White N.J. (1999). Skeletal muscle involvement in falciparum malaria: Biochemical and ultrastructural study. Clin. Infect. Dis..

[11] Vasquez M., Zuniga M., Rodriguez A. (2021). Oxidative Stress and Pathogenesis in Malaria. Front. Cell. Infect. Microbiol..

[12] Olanlokun J.O., Balogun F.A., Olorunsogo O.O. (2021). Chemotherapeutic and prophylactic antimalarial drugs induce cell death through mitochondrial-mediated apoptosis in murine models. Drug Chem. Toxicol..

[13] Cloonan S.M., Choi A.M. (2012). Mitochondria: Commanders of innate immunity and disease?. Curr. Opin. Immunol..

[14] Arnoult D., Soares F., Tattoli I., Girardin S.E. (2011). Mitochondria in innate immunity. EMBO Rep..

[15] West A.P., Shadel G.S., Ghosh S. (2011). Mitochondria in innate immune responses. Nat. Rev. Immunol..

[16] Chen Y., Lu H., Liu Q., Huang G., Lim C.P., Zhang L., Hao A., Cao X. (2012). Function of GRIM-19, a mitochondrial respiratory chain complex I protein, in innate immunity. J. Biol. Chem..

[17] Oreagba I.A., Oshikoya K.A., Amachree M. (2011). Herbal medicine use among urban residents in Lagos, Nigeria. BMC Complement. Altern. Med..

[18] Olanlokun J.O., Babarinde C.O., Olorunsogo O.O. (2020). Antimalarial properties and preventive effects on mitochondrial dysfunction by extract and fractions of *Phyllanthus amarus* (Schum. and Thonn) in *Plasmodium berghei*-infected mice. J. Basic Clin. Physiol. Pharmacol..

[19] Dawurung C.J., Nguyen M.T.H., Pengon J., Dokladda K., Bunyong R., Rattanajak R., Kamchonwongpaisan S., Nguyen P.T.M., Pyne S.G. (2021). Isolation of bioactive compounds from medicinal plants used in traditional medicine: Rautandiol B, a potential lead compound against *Plasmodium falciparum*. BMC Complement. Med. Ther..

[20] Laryea M.K., Sheringham Borquaye L. (2021). Antimalarial, Antioxidant, and Toxicological Evaluation of Extracts of *Celtis africana*, *Grosseria vignei*, *Physalis micrantha*, and *Stachytarpheta angustifolia*. Biochem. Res. Int..

[21] Appiah-Opong R., Agyemang K., Dotse E., Atchoglo P., Owusu K.B., Aning A., Sakyiamah M., Adegle R., Ayertey F., Appiah A.A. (2022). Anti-plasmodial, Cytotoxic and Antioxidant Activities of Selected Ghanaian Medicinal Plants. J. Evid. Based Integr. Med..

[22] Shah K.A., Patel M.B., Patel R.J., Parmar P.K. (2010). *Mangifera indica* (mango). Pharmacogn. Rev..

[23] Okello D., Kang Y. (2019). Exploring Antimalarial Herbal Plants across Communities in Uganda Based on Electronic Data. Evid. Based Complement. Altern. Med..

[24] Mathew L.S., Peter E.L., Weisheit A., Tolo C.U., Deng A.L., Ogwang P.E. (2021). Ethno medical knowledge and traditional use of *Aristolochia bracteolata* Lam. for malaria among local communities in Jubek State of South Sudan: A cross-sectional survey. J. Ethnopharmacol..

[25] Talontsi F.M., Lamshoft M., Douanla-Meli C., Kouam S.F., Spiteller M. (2014). Antiplasmodial and cytotoxic dibenzofurans from *Preussia* sp. harboured in *Enantia chlorantha* Oliv. Fitoterapia.

[26] Babalola A.S., Idowu O.A., Ademolu K.O., Olukunle J., Rahman S.A. (2020). Antiplasmodial activities and abortifacient properties of three commonly used African indigenous anti-malarial plants in *Plasmodium berghei* infected pregnant mice: Implication for maternal and fetal health. Bull. Natl. Res. Cent..

[27] Mbah C.C., Akuodor G.C., Anyalewechi N.A., Iwuanyanwu T.C., Osunkwo U.A. (2012). In vivo antiplasmodial activities of aqueous extract of *Bridelia ferruginea* stem bark against *Plasmodium berghei berghei* in mice. Pharm. Biol..

[28] Lehane A.M., Saliba K.J. (2008). Common dietary flavonoids inhibit the growth of the intraerythrocytic malaria parasite. BMC Res. Notes.

[29] Bhatt D., Kumar S., Kumar P., Bisht S., Kumar A., Maurya A.K., Pal A., Bawankule D.U. (2022). Rutin ameliorates malaria pathogenesis by modulating inflammatory mechanism: An in vitro and in vivo study. Inflammopharmacology.

[30] Barliana M.I., Suradji E.W., Abdulah R., Diantini A., Hatabu T., Nakajima-Shimada J., Subarnas A., Koyama H. (2014). Antiplasmodial properties of kaempferol-3-O-rhamnoside isolated from the leaves of *Schima wallichii* against chloroquine-resistant *Plasmodium falciparum*. Biomed. Rep..

[31] Ganesh D., Fuehrer H.P., Starzengruber P., Swoboda P., Khan W.A., Reismann J.A., Mueller M.S., Chiba P., Noedl H. (2012). Antiplasmodial activity of flavonol quercetin and its analogues in *Plasmodium falciparum*: Evidence from clinical isolates in Bangladesh and standardized parasite clones. Parasitol. Res..

[32] Ali A.H., Sudi S., Shi-Jing N., Hassan W.R.M., Basir R., Agustar H.K., Embi N., Sidek H.M., Latip J. (2021). Dual Anti-Malarial and GSK3beta-Mediated Cytokine-Modulating Activities of Quercetin Are Requisite of Its Potential as a Plant-Derived Therapeutic in Malaria. Pharmaceuticals.

[33] Jin H., Xu Z., Cui K., Zhang T., Lu W., Huang J. (2014). Dietary flavonoids fisetin and myricetin: Dual inhibitors of *Plasmodium falciparum* falcipain-2 and plasmepsin II. Fitoterapia.

[34] Boniface P.K., Ferreira E.I. (2019). Flavonoids as efficient scaffolds: Recent trends for malaria, leishmaniasis, Chagas disease, and dengue. Phytother. Res..

[35] Kumar S., Christophides G.K., Cantera R., Charles B., Han Y.S., Meister S., Dimopoulos G., Kafatos F.C., Barillas-Mury C. (2003). The role of reactive oxygen species on *Plasmodium* melanotic encapsulation in *Anopheles gambiae*. Proc. Natl. Acad. Sci. USA.

[36] Ofori E.A., Garcia-Senosiain A., Naghizadeh M., Kana I.H., Dziegiel M.H., Adu B., Singh S., Theisen M. (2023). Human blood neutrophils generate ROS through FcγR-signaling to mediate protection against febrile *P. falciparum* malaria. Commun. Biol..

[37] Falade C.O., Ajayi I.O., Nsungwa-Sabiiti J., Siribie M., Diarra A., Serme L., Afonne C., Yusuf O.B., Gansane Z., Jegede A.S. (2016). Malaria Rapid Diagnostic Tests and Malaria Microscopy for Guiding Malaria Treatment of Uncomplicated Fevers in Nigeria and Prereferral Cases in 3 African Countries. Clin. Infect. Dis..

[38] Malann Y.D., Matur B.M., Akinnagbe E.S. (2014). Antiplasmodial Activity of Extracts and Fractions of *Mangifera indica* Against *Plasmodium berghei*. Niger. J. Parasitol..

[39] Das N.G., Rabha B., Talukdar P.K., Goswami D., Dhiman S. (2016). Preliminary in vitro antiplasmodial activity of *Aristolochia griffithii* and *Thalictrum foliolosum* DC extracts against malaria parasite *Plasmodium falciparum*. BMC Res. Notes.

[40] Agbaje E.O., Onabanjo A.O. (1991). The effects of extracts of *Enantia chlorantha* in malaria. Ann. Trop. Med. Parasitol..

[41] Olanlokun J.O., Olotu A.F., David O.M., Idowu T.O., Soliman E.M., Olorunsogo O.O. (2019). A novel compound purified from *Alstonia boonei* inhibits *Plasmodium falciparum* lactate dehydrogenase and plasmepsin II. J. Biomol. Struct. Dyn..

[42] Akinmoladun A.C., Ibukun E.O., Afor E., Akinrinlola B.L., Onibon T.R., Akinboboye A.O., Obuotor E.M., Farombi E.O. (2007). Chemical constituents and antioxidant activity of *Alstonia boonei*. Afr. J. Biotechnol..

[43] Moore L.R., Fujioka H., Williams P.S., Chalmers J.J., Grimberg B., Zimmerman P., Zborowsk M.I. (2013). Hemoglobin degradation in malaria-infected erythrocytes determined from live cell magnetophoresis. FASEB J..

[44] Kotepui M., Phunphuech B., Phiwklam N., Chupeerach C., Duangmano S. (2014). Effect of malarial infection on haematological parameters in population near Thailand-Myanmar border. Malar. J..

[45] Guha M., Kumar S., Choubey V., Maity P., Bandyopadhyay U. (2006). Apoptosis in liver during malaria: Role of oxidative stress and implication of mitochondrial pathway. FASEB J..

[46] Olanlokun J.O., Owolabi A.B., Odedeyi A., Oderinde S.O., Bodede O., Steenkamp P., Koorbanally N.A., Olorunsogo O.O. (2024). Mechanism of antimalarial action and mitigation of infection-mediated mitochondrial dysfunction by phyto-constituents of *Andrographis paniculata* ((Burm f.) Wall. ex Nees) in *Plasmodium berghei*-infected mice. J. Ethnopharmacol..

[47] Webster K.A. (2012). Mitochondrial membrane permeabilization and cell death during myocardial infarction: Roles of calcium and reactive oxygen species. Future Cardiol..

[48] Oludele O.J., Adisa B.A., Olufunso O.O. (2020). Regulated rutin co-administration reverses mitochondrial-mediated apoptosis in *Plasmodium berghei*-infected mice. Biochem. Biophys. Res. Commun..

[49] Xu C., Hwang W., Jeong D.E., Ryu Y., Ha C.M., Lee S.V., Liu L., He Z.M. (2018). Genetic inhibition of an ATP synthase subunit extends lifespan in *C. elegans*. Sci. Rep..

[50] Joshi Y.K., Tandon B.N., Acharya S.K., Babu S., Tandon M. (1986). Acute hepatic failure due to *Plasmodium falciparum* liver injury. Liver.

[51] Devarbhavi H., Alvares J.F., Kumar K.S. (2005). Severe falciparum malaria simulating fulminant hepatic failure. Mayo Clin. Proc..

[52] Ali M., Khan T., Fatima K., Ali Q.U.A., Ovais M., Khalil A.T., Ullah I., Raza A., Shinwari Z.K., Idrees M. (2018). Selected hepatoprotective herbal medicines: Evidence from ethnomedicinal applications, animal models, and possible mechanism of actions. Phytother. Res..

[53] Wan L., Jiang J.-G. (2018). Protective effects of plant-derived flavonoids on hepatic injury. J. Funct. Foods.

[54] Li X., Jin Q., Yao Q., Xu B., Li L., Zhang S., Tu C. (2018). The Flavonoid Quercetin Ameliorates Liver Inflammation and Fibrosis by Regulating Hepatic Macrophages Activation and Polarization in Mice. Front. Pharmacol..

[55] Ilyas U., Katare D.P., Aeri V., Naseef P.P. (2016). A Review on Hepatoprotective and Immunomodulatory Herbal Plants. Pharmacogn. Rev..

[56] Farghali H., Kamenikova L., Hynie S., Kmonickova E. (2000). Silymarin effects on intracellular calcuim and cytotoxicity: A study in perfused rat hepatocytes after oxidative stress injury. Pharmacol. Res..

[57] Bolchoz L.J., Gelasco A.K., Jollow D.J., McMillan D.C. (2002). Primaquine-induced hemolytic anemia: Formation of free radicals in rat erythrocytes exposed to 6-methoxy-8-hydroxylaminoquinoline. J. Pharmacol. Exp. Ther..

[58] Gopalakrishnan A.M., Kumar N. (2015). Antimalarial action of artesunate involves DNA damage mediated by reactive oxygen species. Antimicrob. Agents Chemother..

[59] Haynes R.K., Krishna S. (2004). Artemisinins: Activities and actions. Microbes Infect..

[60] Pandey A.V., Bisht H., Babbarwal V.K., Srivastava J., Pandey K.C., Chauhan V.S. (2001). Mechanism of malarial haem detoxification inhibition by chloroquine. Biochem. J..

[61] Ty M.C., Zuniga M., Gotz A., Kayal S., Sahu P.K., Mohanty A., Mohanty S., Wassmer S.C., Rodriguez A. (2019). Malaria inflammation by xanthine oxidase-produced reactive oxygen species. EMBO Mol. Med..

[62] Mahittikorn A., Kwankaew P., Rattaprasert P., Kotepui K.U., Masangkay F.R., Kotepui M. (2022). Elevation of serum interleukin-1beta levels as a potential indicator for malarial infection and severe malaria: A meta-analysis. Malar. J..

[63] Kotepui K.U., Mahittikorn A., Wilairatana P., Masangkay F.R., Kotepui M. (2023). Association between Plasmodium Infection and Nitric Oxide Levels: A Systematic Review and Meta-Analysis. Antioxidants.

[64] Agina A.A., Abd-Allah S.H. (1999). Plasma levels of nitric oxide in association with severe *Plasmodium falciparum* in Yemen. J. Egypt. Soc. Parasitol..

[65] Weinberg J.B., Lopansri B.K., Mwaikambo E., Granger D.L. (2008). Arginine, nitric oxide, carbon monoxide, and endothelial function in severe malaria. Curr. Opin. Infect. Dis..

[66] Weinberg J.B., Volkheimer A.D., Rubach M.P., Florence S.M., Mukemba J.P., Kalingonji A.R., Langelier C., Chen Y., Bush M., Yeo T.W. (2016). Monocyte polarization in children with falciparum malaria: Relationship to nitric oxide insufficiency and disease severity. Sci. Rep..

[67] Sarr D., Oliveira L.J., Russ B.N., Owino S.O., Middii J.D., Mwalimu S., Ambasa L., Almutairi F., Vulule J., Rada B. (2021). Myeloperoxidase and Other Markers of Neutrophil Activation Associate With Malaria and Malaria/HIV Coinfection in the Human Placenta. Front. Immunol..

[68] Boulet C., Gaynor T.L., Carvalho T.G. (2021). Eryptosis and Malaria: New Experimental Guidelines and Re-Evaluation of the Antimalarial Potential of Eryptosis Inducers. Front. Cell Infect. Microbiol..

[69] Naaz F., Javed S., Abdin M.Z. (2007). Hepatoprotective effect of ethanolic extract of *Phyllanthus amarus* Schum. et Thonn. on aflatoxin B1-induced liver damage in mice. J. Ethnopharmacol..

[70] Zimmerman H.J. (1999). Hepatotoxicity: The Adverse Effects of Drugs and Other Chemicals on the Liver.

[71] Thorogood N., Atwal S., Mills W., Jenner M., Lewis D.A., Cavenagh J.D., Agrawal S.G. (2007). The risk of antimalarials in patients with renal failure. Postgrad. Med. J..

[72] IARC Working Group on the Evaluation of Carcinogenic Risks to Humans (1987). 8-Methoxypsoralen (Methoxsalen) Plus Ultraviolet Radiation (Group 1). Overall Evaluations of Carcinogenicity: An Updating of IARC Monographs Volumes 1 to 42.

[73] Howard M.A., Hibbard A.B., Terrell D.R., Medina P.J., Vesely S.K., George J.N. (2003). Quinine allergy causing acute severe systemic illness: Report of 4 patients manifesting multiple hematologic, renal, and hepatic abnormalities. Bayl. Univ. Med. Cent. Proc..

[74] Okhale S.E., Egharevba H.O., Okpara O.J., Ugbabe G.E., Ibrahim J.A., Fatokum O.T., Sulyman A.O., Igoli J.O. (2019). Aristolochic acids in herbal medicine: Public health concerns for consumption and poor regulation of botanical products in Nigeria and West Africa. J. Med. Plants Res..

[75] Grollman A.P., Marcus D.M. (2016). Global hazards of herbal remedies: Lessons from *Aristolochia*: The lesson from the health hazards of *Aristolochia* should lead to more research into the safety and efficacy of medicinal plants. EMBO Rep..

[76] Jhuang J.R., Chiang C.J., Su S.Y., Yang Y.W., Lee W.C. (2019). Reduction in the Incidence of Urological Cancers after the Ban on Chinese Herbal Products Containing Aristolochic Acid: An Interrupted Time-Series Analysis. Sci. Rep..

[77] Wang C., Liu Y., Han J., Li W., Sun J., Wang Y. (2023). Detection and Removal of Aristolochic Acid in Natural Plants, Pharmaceuticals, and Environmental and Biological Samples: A Review. Molecules.

[78] Green C.J., Hodson L. (2014). The influence of dietary fat on liver fat accumulation. Nutrients.

[79] Patel S.R., Hartwig J.H., Italiano J.E. (2005). The biogenesis of platelets from megakaryocyte proplatelets. J. Clin. Investig..

[80] O’Sullivan J.M., O’Donnell J.S. (2018). Platelets in malaria pathogenesis. Blood.

[81] Ajewole O.I., Fasoro O. (2013). Market and marketing information of Bodija plank market in Ibadan metropolis, Ibadan, Oyo State, Nigeria. Niger. J. For..

[82] Hudzicki J., Kirby-Bauer Disk Diffusion Susceptibility Test Protocol (2009). American Society for Microbiology. https://asm.org/protocols/kirby-bauer-disk-diffusion-susceptibility-test-pro.

[83] Makler M.T., Hinrichs D.J. (1993). Measurement of the lactate dehydrogenase activity of *Plasmodium falciparum* as an assessment of parasitemia. Am. J. Trop. Med. Hyg..

[84] Ryley J.F., Peters W. (1970). The antimalarial activity of some quinolone esters. Ann. Trop. Med. Parasitol..

[85] Asakura T., Minakata K., Adachi K., Russell M.O., Schwartz E. (1977). Denatured hemoglobin in sickle erythrocytes. J. Clin. Investig..

[86] Johnson D., Lardy H. (1967). [15] Isolation of liver or kidney mitochondria. Methods in Enzymology.

[87] Lowry O.H., Rosebrough N.J., Farr A.L., Randall R.J. (1951). Protein measurement with the Folin phenol reagent. J. Biol. Chem..

[88] Lapidus R.G., Sokolove P.M. (1993). Spermine Inhibition of the permeability transition of isolated rat liver mitochondria: An investigation of mechanisms. Biochem. Biophys. J..

[89] Lardy H.A., Wellman H. (1953). The catalyst effect of 2,4 dinitrophenol on adeno-sinetriphosphate hydrolysis by cell particles and soluble enzymes. J. Biol. Chem..

[90] Misra H.P., Fridovich I. (1972). The role of superoxide anion in the autoxidation of epinephrine and a simple assay for superoxide dismutase. J. Biol. Chem..

[91] Clairborne A., Greenwald A.R. (1995). Catalase Activity. Handbook of Methods for Oxygen Radical Research.

[92] Rotruck J.T., Pope A.L., Ganther H.E., Swanson A.B., Hafeman D.G., Hoekstra W.G. (1973). Selenium: Biochemical role as a component of glutathione peroxidase. Science.

[93] Beutler E., Duron O., Kelly B.M. (1963). Improved method for the determination of blood glutathione. J. Lab. Clin. Med..

[94] Ohkawa H., Oshishi N., Yagi K. (1979). Assay for lipid peroxidation in animal tissues by Thiobarbituric acid reaction. Anal. Biochem..

[95] Perez-Severiano F., Santamaria A., Pedraza-Chaverri J., Medina-Campos O.N., Rios C., Segovia J. (2004). Increased formation of reactive oxygen species, but no changes in glutathione peroxidase activity, in striata of mice transgenic for the Huntington’s disease mutation. Neurochem. Res..

[96] Owumi S.E., Kazeem A.I., Wu B., Ishokare L.O., Arunsi U.O., Oyelere A.K. (2022). Apigeninidin-rich Sorghum bicolor (L. Moench) extracts suppress A549 cells proliferation and ameliorate toxicity of aflatoxin B1-mediated liver and kidney derangement in rats. Sci Rep.

[97] Trush M.A., Egner P.A., Kensler T.W. (1994). Myeloperoxidase as a biomarker of skin irritation and inflammation. Food Chem. Toxicol..

[98] Bergmeyer H.I., Gawehn K., Grassl M., Bergmeyer H.U. (1974). Methods of Enzymatic Analysis.

