# Supplementary material for: Elucidation of the Active Agents in a West African Ground Herbal Medicine Formulation That Elicit Antimalarial Activities in In Vitro and In Vivo Models
Source: Molecules. 2024 Nov 29;29(23):5658. doi: 10.3390/molecules29235658 (PMC11643963; doi:10.3390/molecules29235658)
Supplement: Supplementary file 1 [file molecules-29-05658-s001.zip › molecules-3289849-supplementary.pptx]

## Slide 1
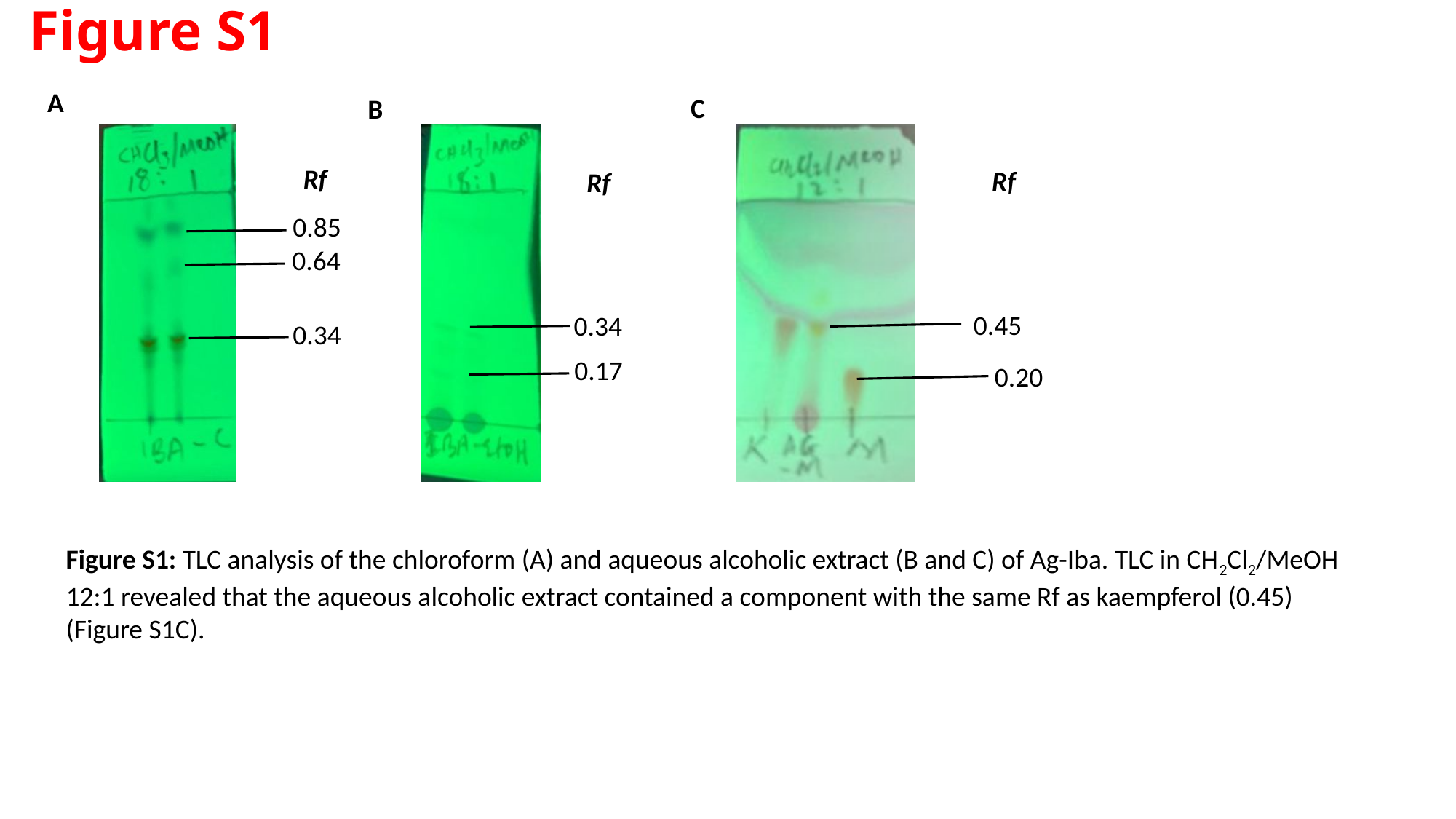

Figure S1
A
C
B
Rf
Rf
Rf
0.85
0.64
0.45
0.34
0.34
0.17
0.20
Figure S1: TLC analysis of the chloroform (A) and aqueous alcoholic extract (B and C) of Ag-Iba. TLC in CH2Cl2/MeOH 12:1 revealed that the aqueous alcoholic extract contained a component with the same Rf as kaempferol (0.45)(Figure S1C).

## Slide 2
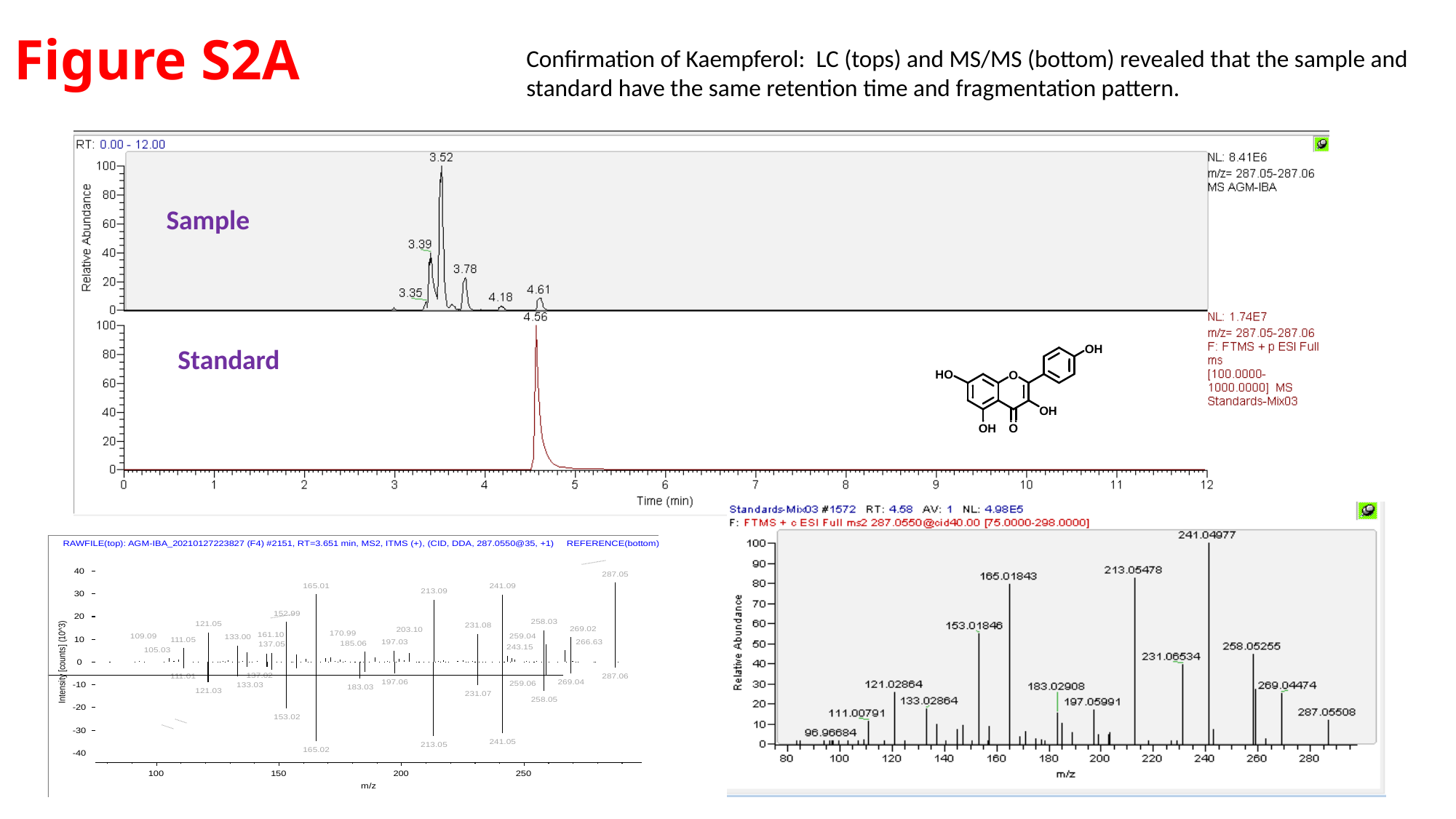

Figure S2A
Confirmation of Kaempferol: LC (tops) and MS/MS (bottom) revealed that the sample and standard have the same retention time and fragmentation pattern.
Sample
Standard

## Slide 3
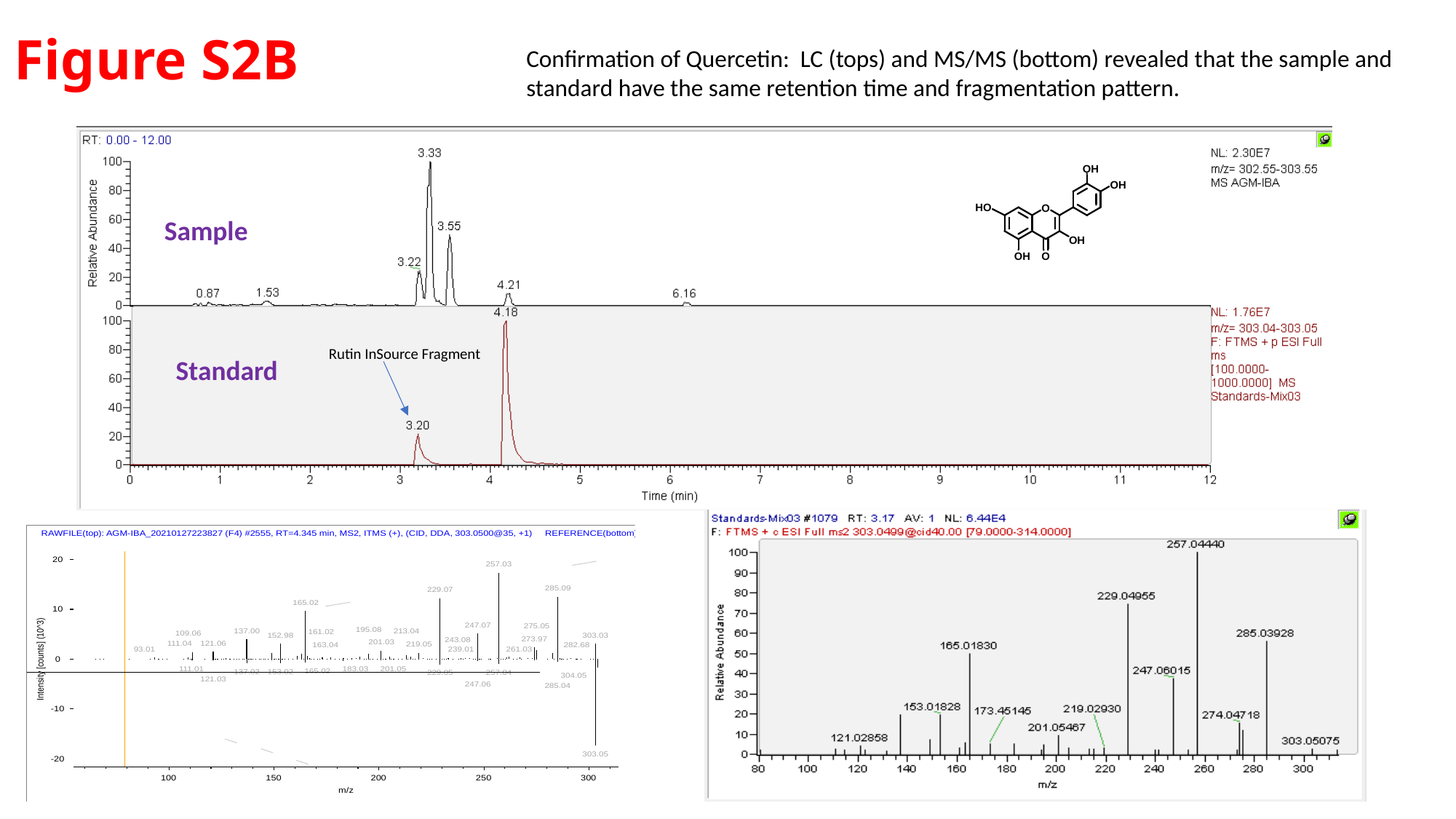

Figure S2B
Confirmation of Quercetin: LC (tops) and MS/MS (bottom) revealed that the sample and standard have the same retention time and fragmentation pattern.
Sample
Rutin InSource Fragment
Standard

## Slide 4
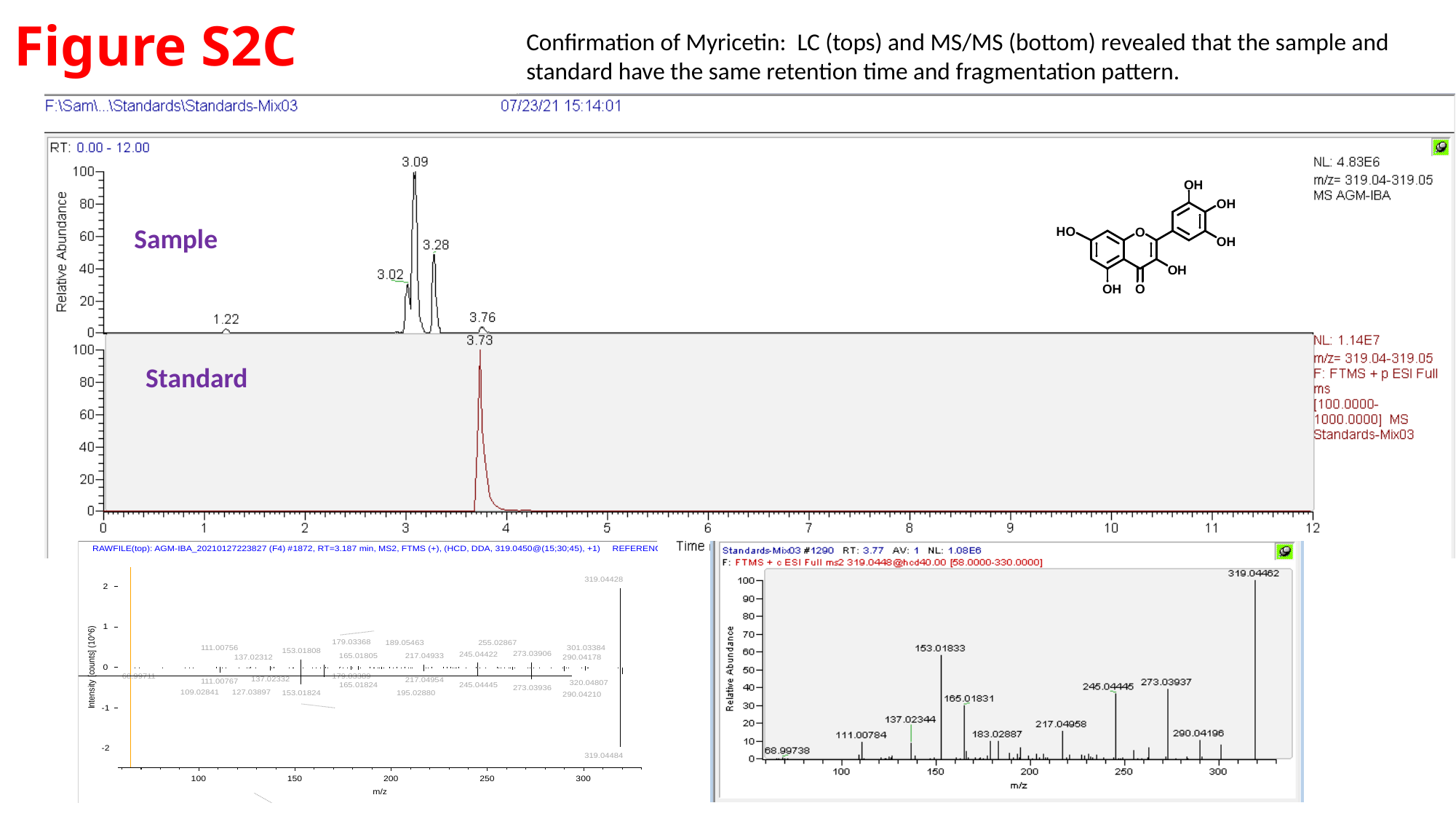

Figure S2C
Confirmation of Myricetin: LC (tops) and MS/MS (bottom) revealed that the sample and standard have the same retention time and fragmentation pattern.
Sample
Standard

## Slide 5
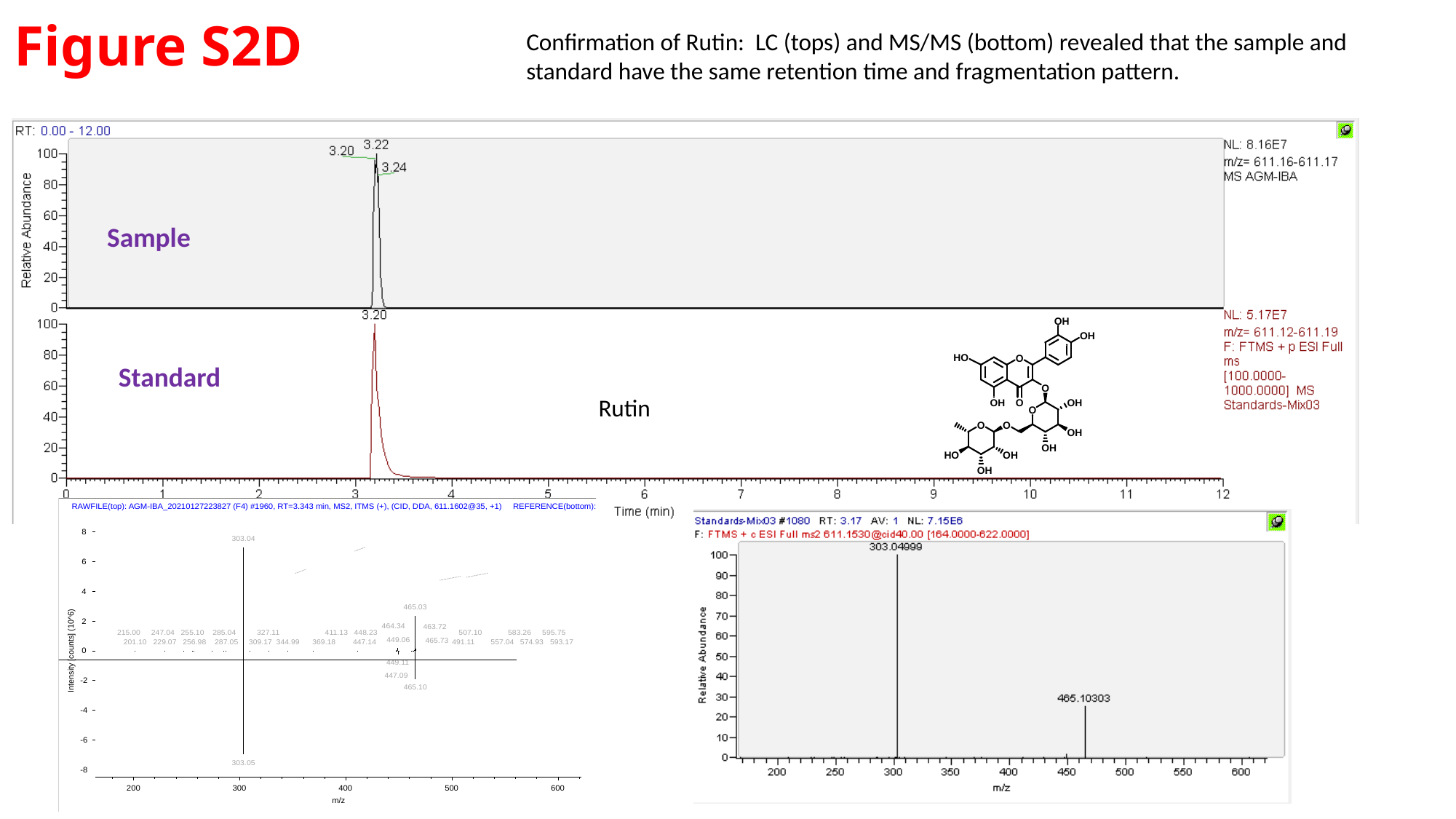

Figure S2D
Confirmation of Rutin: LC (tops) and MS/MS (bottom) revealed that the sample and standard have the same retention time and fragmentation pattern.
Sample
Standard
Rutin

## Slide 6
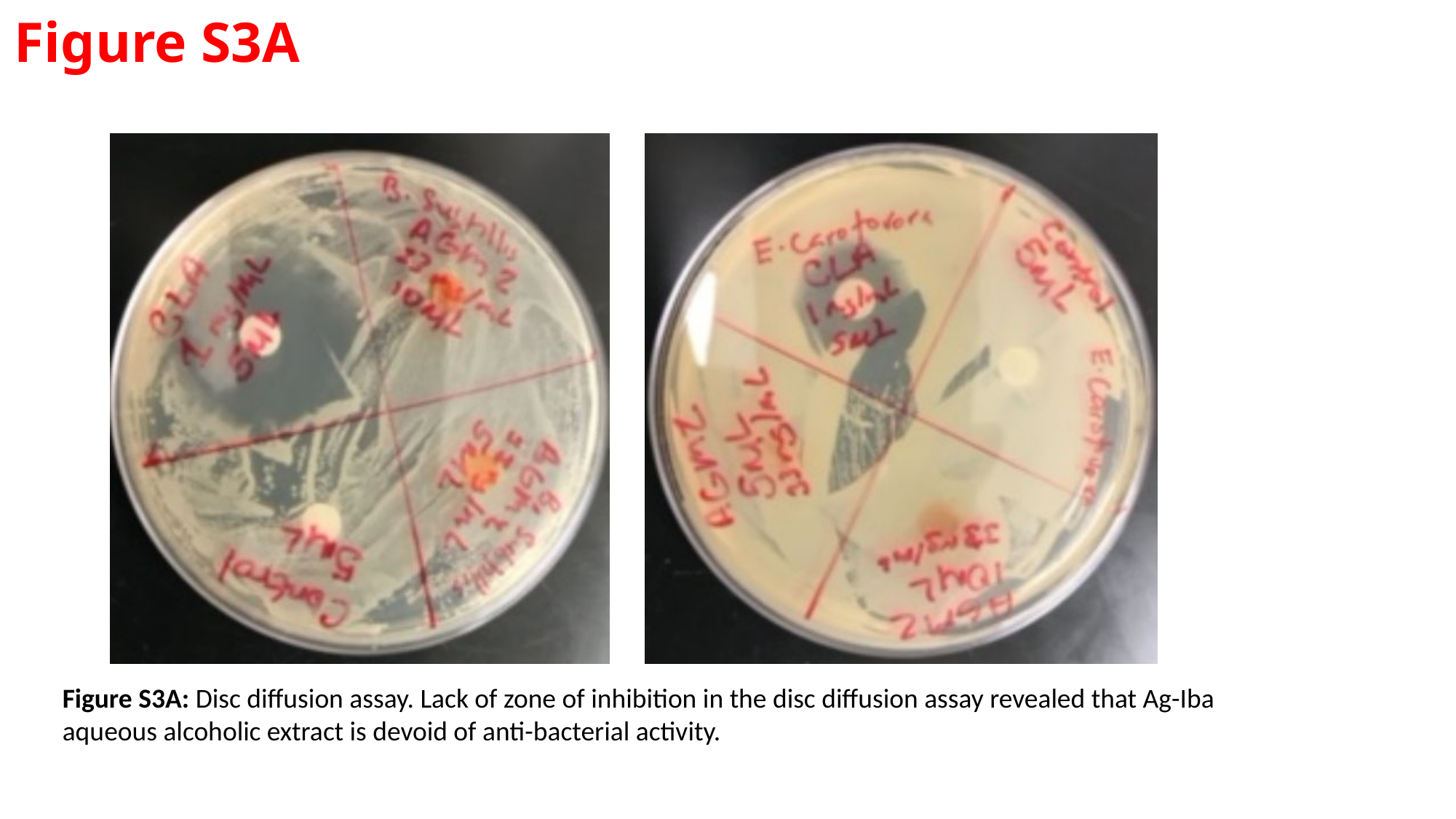

Figure S3A
Figure S3A: Disc diffusion assay. Lack of zone of inhibition in the disc diffusion assay revealed that Ag-Iba aqueous alcoholic extract is devoid of anti-bacterial activity.

## Slide 7
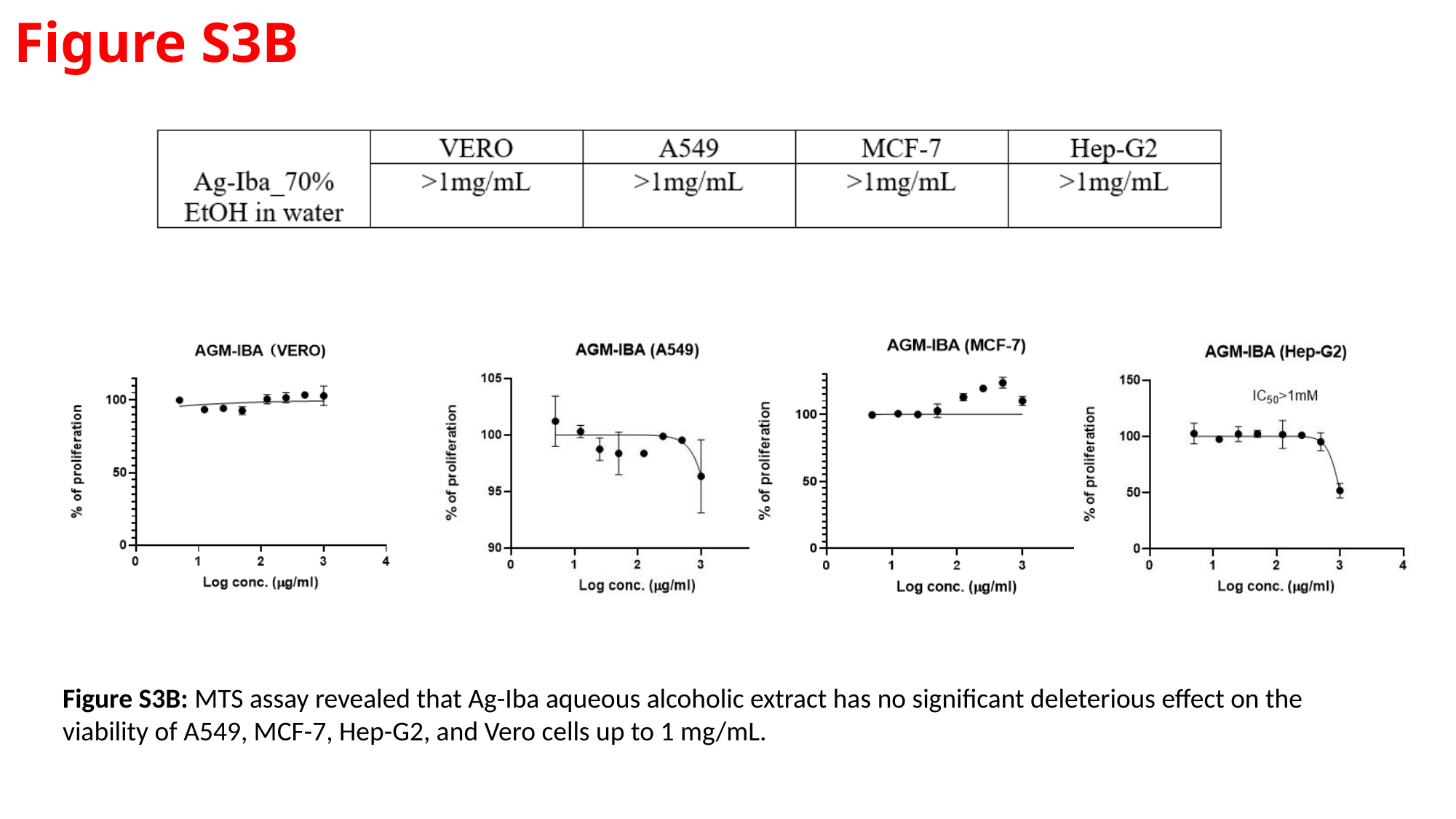

Figure S3B
Figure S3B: MTS assay revealed that Ag-Iba aqueous alcoholic extract has no significant deleterious effect on the viability of A549, MCF-7, Hep-G2, and Vero cells up to 1 mg/mL.
